# Functional and phenotypic characterization of peripheral blood mononuclear cells from tuberculosis patients in Southern Thailand

**DOI:** 10.3389/fimmu.2025.1639808

**Published:** 2025-10-07

**Authors:** Pyae Sone Oo, Jomkwan Ongarj, Ratchanon Sophonmanee, Narisa Mohthong, Sahasawat Suksan, Natapohn Saowaphong, Rachel Tanner, Nawamin Pinpathomrat

**Affiliations:** ^1^ Department of Biomedical Sciences and Biomedical Engineering, Faculty of Medicine, Prince of Songkla University, Songkhla, Thailand; ^2^ Department of Biology, University of Oxford, Oxford, United Kingdom

**Keywords:** Tuberculosis, mycobacterial growth control, MGIA, CD4+ T cells, TNF-α, immune response

## Abstract

**Introduction:**

Tuberculosis (TB) remains a global health challenge, with active TB disease (ATB) and latent TB infection (LTBI) representing distinct immunological states. Understanding immune responses in these groups is critical for developing effective interventions. The complex nature of immune responses to *Mycobacterium tuberculosis* (*M.tb*) within and between different stages of TB, host evasion mechanisms of the bacterium, variable protection conferred by the BCG vaccine in adults, and lack of validated immune correlates of protection are among the key challenges to the successful control of TB.

**Methods:**

In the present study, we conducted functional and phenotypic characterization of peripheral blood mononuclear cells (PBMCs) from a cohort in Southern Thailand. We compared immune responses in individuals with ATB, LTBI and healthy controls (HC) using flow cytometry (ATB n = 9, LTBI n = 11, HC n = 10) and the mycobacterial growth inhibition assay (MGIA) (ATB n = 13, LTBI n = 15, HC n = 15).

**Results:**

MGIA revealed significantly enhanced control of BCG growth in the ATB group compared to LTBI and HC groups. Furthermore, NK cell frequency and TNF-α levels were significantly elevated in ATB compared to LTBI and HC groups, and CD4+ T cell TNF-α responses correlated with mycobacterial growth control.

**Discussion:**

The findings from this study demonstrate differential immune responses across TB stages in this cohort, identify potential cellular markers for TB diagnosis and monitoring, and may guide vaccine strategies and host-directed therapies.

## 1 Introduction

Tuberculosis (TB), caused by *Mycobacterium tuberculosis* (*M.tb*), remains a global public health emergency as defined by the World Health Organisation (WHO). An estimated 8.2 million new TB cases and 1.25 million deaths were reported in 2023 (1, 2). Despite the availability of antibiotics and the BCG vaccine, shortcomings in both approaches mean that TB is still the leading cause of death due to a single infectious agent. Approximately 2 billion people are latently infected with *M.tb*. While active TB disease (ATB) is clinically symptomatic, latent *M.tb* infection (LTBI) is defined as a state of persistent immune response to stimulation by specific antigens with no evidence of clinically manifested active TB; there is no gold standard for the diagnosis of LTBI (3). The immune response in TB is highly heterogeneous and depends on many factors including geography, genetic variation, vaccination history, age and immune status, infection and co-infections, strain diversity, immune modulation by *M.tb*, and disease stage.

The immune characteristics underlying TB disease states are complex and not yet fully understood, despite extensive research. This complexity continues to pose a significant challenge to the development of safe and effective anti-TB vaccines. *M.tb*, unlike many other pathogens, lacks a well-defined immune correlate of protection (CoP) due to its reliance on cell-mediated immunity (CMI), the absence of any validated single marker for protection, lack of a clear immune threshold, variable protective efficacy of the BCG vaccine, and variability in host and pathogen factors (4–7). Previous study have demonstrated that T lymphocytes, especially Th1 and IFN-γ responses, play a central role in immunity against TB but may not be sufficient for protection (8). The direct mycobacterial growth inhibition assay (MGIA) offers an *ex vivo* functional measure of mycobacterial growth control, bridging the gap between preclinical and clinical studies (2, 7, 9). It quantifies immune function by assessing the ability of peripheral blood mononuclear cells (PBMCs) to restrict or control the growth of live *M.tb* or surrogate mycobacteria, such as *Mycobacterium bovis* Bacillus Calmette-Guérin (BCG). The assay has been applied in a range of different populations, including vaccine trials and observational cohorts as a surrogate of protective efficacy and to identify potential immune correlates of protection (2, 10, 11). However, its application in TB-endemic settings, particularly in Southeast Asia, remains limited. These assays are pivotal in TB research and development. Integrating MGIA with flow cytometry in our cohort offers the ability to link functional control of mycobacterial growth with specific immune correlates at the cellular level.

In this study, we employed flow cytometry to profile T cell subsets in PBMCs from an understudied population in Southern Thailand following stimulation with purified protein derivative (PPD). This approach allowed us to characterize the activation and differentiation of CD4+ and CD8+ T cells, as well as CD56+ NK cells and CD3+CD56+ NKT cells across the ATB, LTBI, and HC groups. Moreover, we utilized the MGIA to evaluate the functional ability of PBMCs to control the growth of mycobacteria. Our findings contribute to the body of literature indicating potential immune correlates of protection or biomarkers which may be relevant for the development of a safe and effective TB vaccine.

## 2 Materials and methods

### 2.1 Clinical samples

#### 2.1.1 Clinical study design

The clinical study was conducted in the Department of Biomedical Sciences and Biomedical Engineering, Faculty of Medicine, Prince of Songkla University, Hat Yai, Thailand. The study complied with the declaration of Helsinki (2008) and was approved by the Human Research Ethics Committee (HREU) of Songklanagarind Hospital, Thailand (REC.66-279-38-1).

#### 2.1.2 Inclusion and exclusion criteria

A total of 60 participants ≥18 years of age were enrolled, and all provided written informed consent. Healthy volunteers with a visible BCG vaccination scar on the left arm were enrolled into the HC group. Other inclusion criteria for the HC group were: the absence of any current or past TB-related symptoms, no history of *M.tb* infection or TB treatment, no recent contact with active TB cases, and a negative Tuberculin Skin Test (TST) result. The inclusion criteria for the LTBI group were: individuals at high risk of exposure to *M.tb*, including healthcare workers working with TB patients, living in a high TB prevalence area, and recent close contact with a person diagnosed with TB. In addition, they were required to provide evidence of infection, defined as either TST positive with an induration of ≥ 10 mm in size or a positive interferon-gamma release assay (IGRA). Inclusion criteria for enrolment into the ATB group were: clinical symptoms suggestive of acute TB or confirmed active TB by chest X-ray (CXR)/CT scan accompanied by laboratory investigations with sputum Acid Fast Bacilli (AFB) smear, culture, and Polymerase Chain Reaction (PCR). Participants with known underlying immunodeficiency conditions/diseases were excluded from the study.

**Figure 1:**
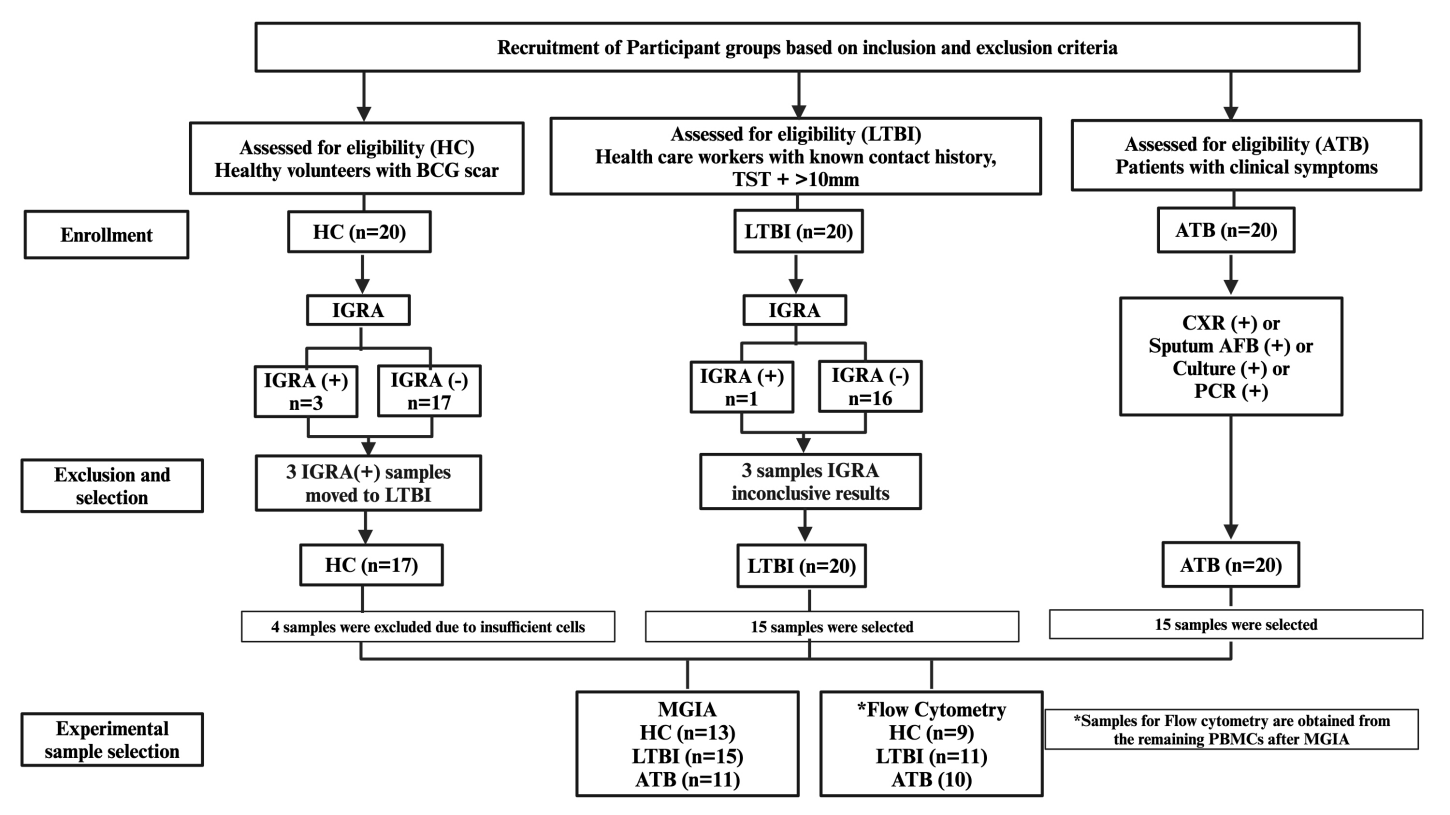
Flowchart of participant recruitment, classification, and sample selection for MGIA and flow cytometry. Three HC with positive IGRA were reclassified as LTBI; three LTBI participants had inconclusive IGRA results but remained classified as LTBI. Samples with insufficient PBMC counts were excluded before analysis. Final MGIA sample sizes: HC = 13, LTBI = 15, ATB = 11. Flow cytometry was performed on PBMCs remaining after MGIA (*HC = 9, LTBI = 11, ATB = 10). ATB, active tuberculosis; LTBI, latent tuberculosis infection; HC, healthy control; IGRA, interferon-γ release assay; TST, tuberculin skin test; CXR, chest X-ray; AFB, acid-fast bacilli; PCR, polymerase chain reaction.

20 HC, 20 LTBI, and 20 ATB subjects were enrolled. Participants from the HC and LTBI groups received an IGRA test by QuantiFERON-TB Gold Plus (QFT-Plus, Qiagen, Germany) following manufacturer’s instructions. Of the 20 HC samples, 3 were IGRA (+) and were reallocated to the LTBI group to avoid misclassification, and 17 were IGRA (-) of which 4 samples were excluded due to insufficient cell recovery for the planned assays. From the LTBI group of 20 samples, 1 was IGRA (+), 3 were inconclusive, and 16 were IGRA (-). The final sample set thus comprised 13 HC, 15 LTBI and 15 ATB, which is in line with calculated requirements for our primary outcome, MGIA, based on previously published data (11). The study flow process is illustrated in **Figure 1**.

#### 2.1.3 Sample collection and processing

Possible risks and complications of venipuncture were explained to participants prior to blood collection. 20 mL of heparinized whole blood was collected from each participant. PBMCs were isolated from fresh heparinized whole blood under aseptic conditions using density gradient centrifugation, as described previously (12). Briefly, whole blood was transferred into a 50 mL high clarity, sterile conical centrifuge tube (Falcon^®^) and centrifuged at 840xg, 22°C for 10 min. After removal and storage of plasma, the remaining blood was diluted with Roswell Park Memorial Institute (RPMI) 1640 with L-glutamine (Gibco, ThermoFisher Scientific). The diluted blood was gently layered onto a conical centrifuge tube preloaded with 20 mL LymphoPrep™ (StemCell Technologies, France). The tubes were centrifuged at 840xg for 30 min, 22°C without break. After centrifugation, the visible white band of PBMCs was transferred into a new falcon tube and topped up with RPMI 1640 to 30 mL, then centrifuged at 840xg for 10 min, 22°C with break. The supernatants were discarded and the pellets of PBMCs were washed with RPMI 1640, then centrifuged 300xg for 10 min, 22°C. The supernatants were discarded, and the pellets were resuspended in 5 mL RPMI 1640. The cells were stained with 0.4% trypan blue dye at a 1:1 dilution for counting on the Countess 3 automated cell counter (Thermo Fisher Scientific). Then, the pellets were resuspended in 20 mL of RPMI 1640 and centrifuged at 470xg for 5 min, 22°C. The supernatant was discarded, and cells were resuspended in pre-chilled freezing media (containing 90% FBS heat inactivated and filtered + 10% DMSO) to give a final concentration of 3 x 10^6^ cells/mL. The cells were aliquoted into cryovials and placed in cell freezing containers (Corning^®^ CoolCell^®^ Containers) which were stored at -80°C for 1–2 days and then transferred to liquid nitrogen until use.

#### 2.1.4 PBMC thawing

Cryopreserved PBMC samples were thawed at 37°C in a water bath and then each sample was gently poured into a 50 mL tube and topped up with 5 mL of pre-chilled R10 medium (RPMI medium + 10% FBS + 1% HEPES) and rested for 10 minutes. The tubes were then centrifuged at 250 xg for 5 minutes at 25°C. The supernatants were removed, and the pellets were washed with 10 mL of R10 media with 10U/mL of benzonase endonuclease. The tubes were incubated with a loose cap at 37°C with CO_2_ for 2 hours. The cells were then washed again with 10 mL of R10 medium and resuspended with 2 mL of R10 before counting using a Countess™ 3, Invitrogen™ automated cell counter.

### 2.2 PBMC immunophenotyping

Cryopreserved PBMCs were seeded into a U-bottom plate at a concentration of 1 x 10^6^ cells/well in a volume of 200 µl/well. The cells were supplemented with R10 (RPMI containing 10% FBS, 2 mM 1-glutamine, 1 mM Sodium Pyruvate, 100U/mL of penicillin and streptomycin) together with CD28 and CD49d co-stimulatory antibodies at a concentration of 1µg/mL each. For the positive control wells, Phorbol 12-Myristate 13-Acetate (PMA)/Ionomycin was added at a volume of 50 µl/well. Unstimulated wells (negative control) and Live/Dead wells were added with R10 medium and plates incubated at 37°C, 5% CO_2_ for 2 hours. After incubation, Brefeldin A at a concentration of 0.2 µg/mL and Monensin at a concentration of 0.1µg/mL; (BD Biosciences) were added and plates incubated at 37°C, 5% CO_2_ for 16 hours. 20 µl of PPD-T (SSI, Denmark) at a final concentration of 20 µg/mL was added as a stimulating antigen to the seeded cells. The cell surface staining panel included anti-CD14-BV510, anti-CD19-BV510, anti-CD56-PE-Cy5, anti-CD4 -APC-Cy7; BioLegend^®^ at a concentration of 0.5 µl, 0.5 µl, 1 µl and 1 µl respectively.

#### 2.2.1 Intracellular cytokine staining

**Figure 2:**
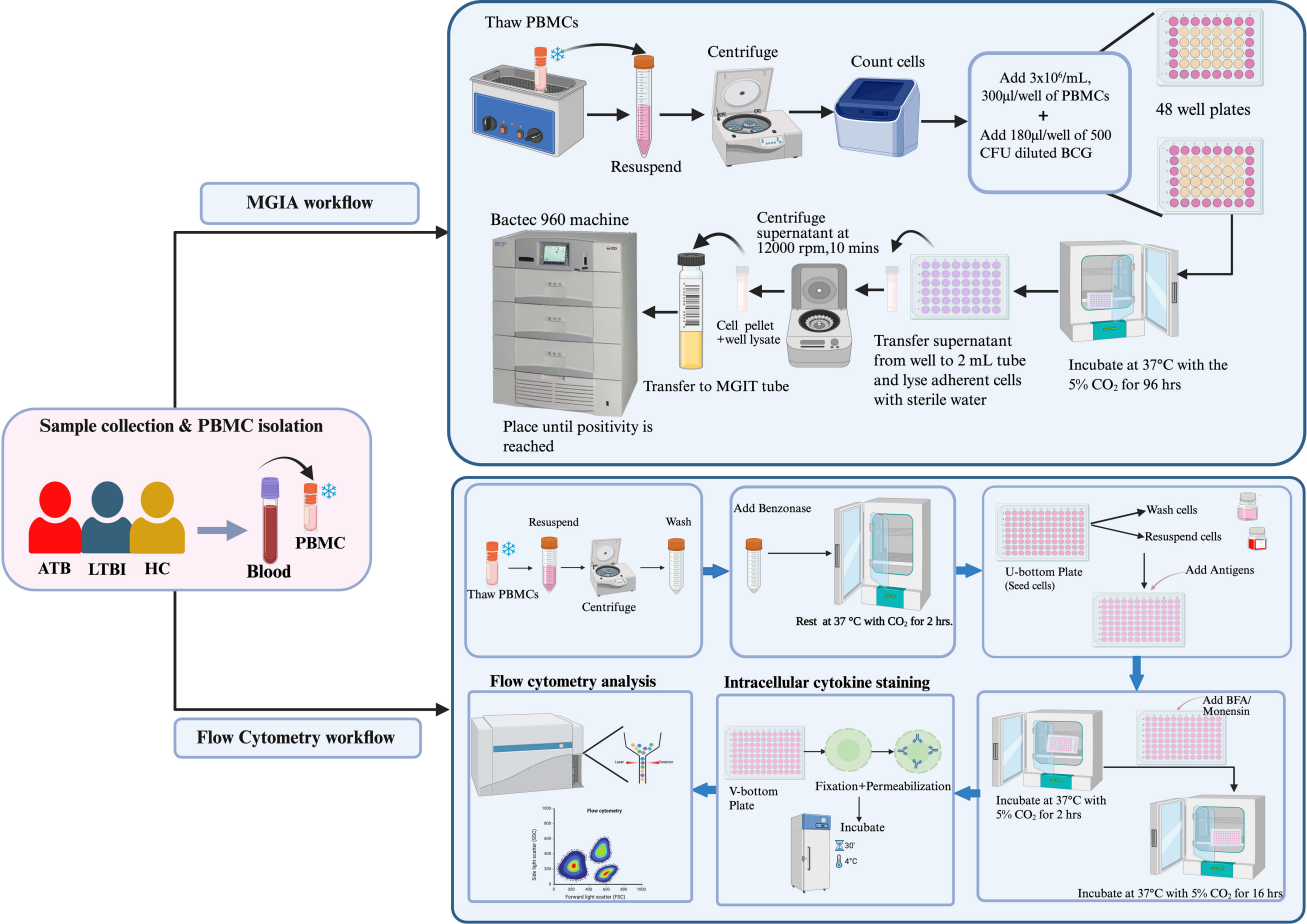
Experimental flowchart of the study, illustrating sample collection and processing, Mycobacterium Growth Inhibition Assay (MGIA), and flow cytometry protocols for PPD stimulation, surface, and intracellular staining.

The cells were centrifuged and transferred to a V-bottom plate the next day and then centrifuged at 470xg at 22°C for 5 minutes. The cells were then harvested by spinning down the plate at 25°C, 1800xg for 3 minutes. The supernatants were discarded, washed with 200 µl of PBS (Invitrogen, Waltham, MA, USA) and centrifuged at 1800xg for 3 minutes. Subsequently, 5 µl of LIVE/DEAD (L/D) Fixable Aqua Dead Cell Stain (ThermoFisher Scientific, Waltham, MA, USA) diluted at 1:100 in PBS was added to the cells and incubated at 4°C in the dark for 10 minutes. 45 µl of surface antibody cocktail diluted in 1% BSA in PBS was added into the wells and then incubated at 4°C in the dark for 30 minutes. Following surface staining, 200 µl of CytoFix (BD Biosciences, NJ, USA) was added to the cells for fixation and permeabilization and incubated at 4°C in the dark for 20 minutes. The plate was washed with 200 µl of CytoPerm (BD Biosciences, NJ, USA) and centrifuged at 1800xg, 4°C for 3 minutes. The fixed cells were stained intracellularly with 50 µl of intracellular cytokine staining (ICS) antibody cocktail comprising anti-CD3-AF700 (Ebioscience), anti-CD8-BV650 (BioLegend), anti-IL-2-PE (BioLegend), anti-IL-4-PE-Dazzle (BioLegend), anti-IL-17-FITC (BioLegend), anti-IFN-γ-PE-Cy7 (Ebioscience), and anti-TNF-α-AF647(BioLegend) and incubated at 4°C in the dark for 30 minutes. After incubation, the cells were washed twice with 200 µl of CytoPerm and centrifuged at 1800xg, 4°C for 3 minutes. The cells were reconstituted with 100 µl of PBS/BSA, mixed well and transferred into labelled cluster tubes. The tubes were sorted using a CytoFLEX S flow cytometer (Beckman Coulter, USA) with CytExpert software (v. 2.5). The output files (FCS) then underwent further downstream analysis using FlowJo (USA) Software (v.10) (**Figure 2**).

### 2.3 Direct PBMC mycobacterial growth inhibition assay

#### 2.3.1 BCG inoculum preparation

The MGIA was tech-transferred to the Prince of Songkla University from developers at the University of Oxford (13) using in-house lyophilized Bacillus Calmette-Guérin (BCG) from Queen Saovabha Memorial Institute strain Tokyo 172-1; BCG (QSMI) which is the standard strain used for vaccination in Thailand. The MGIA assay has previously been described in mice, cattle, non-human primates (NHPs), and human (2, 7, 13–21). Briefly, lyophilized BCG (QSMI) was dissolved in 1 mL of saline and thoroughly mixed. Subsequently, 1 mL of the BCG solution was added to 9 mL of 7H9 broth media (supplemented with 10% OADC, 0.05% Tween 80) and incubated at 37°C with shaking at 170 rpm for 5 days. The 10 mL of inoculum was then transferred to a flask containing 95 mL of 7H9 broth media and further incubated at 37°C with shaking at 170 rpm until the optical density (OD) reached 0.4 at 600 nm. The BCG culture was then collected in a volume of 3 mL, adjusted to 0.5× growth medium with 10% glycerol, and stored at -80°C.

#### 2.3.2 Generation of a standard curve

The frozen BCG stock was thawed at 37°C in a water bath, then sonicated 3 times for 30 seconds with intervening intervals of 30 seconds on ice. Serial dilutions were prepared with Phosphate-Buffered Saline (PBS) containing 0.05% Tween 80, with a 1:10 serial dilution curve made from dilution 1 to 6. In parallel, BACTEC mycobacterium growth inhibition tubes (MGITs) were prepared containing 800 µl of PANTA (Polymyxin B, Amphotericin, Nalidixic acid Trimethoprim, and Azlocillin, BD BBL™ MGIT™) antibiotics and oleic acid, bovine albumin, sodium chloride, dextrose, and catalase (OADC) (BD, BB™ MGIT™) growth enrichment. Subsequently, 500 µl of each dilution was added to the supplemented MGIT tubes and placed in the BACTEC 960 machine (BD Biosciences, USA) until the time to positivity (TTP) was recorded. The remaining volume of each dilution was plated as 25 µl onto 7H10 agar (10% OADC, 0.5% glycerol), divided into quarters. The TTP results were plotted against Colony Forming Units (CFU) on a semi-log line, and subjected to linear regression analysis. The equation describing the line was solved for X using the formula: Y = A*X+B to X = (Y-B)/A, where A represents the slope and B, the y-intercept (**Supplementary Figure S1**).

#### 2.3.3 MGIA optimization

Cryopreserved PBMCs were thawed in a 37°C water bath, then added to 10 mL of RPMI containing L-glutamine, 10% Foetal Bovine Serum (FBS), and 25 mM HEPES before centrifuging at 250 xg for 7 minutes. Subsequently, the cells were resuspended in the same media supplemented with 2 µl/mL of 25U Benzonase, and allowed to rest at 37°C with CO_2_ for 2 hours before counting. During the incubation period, the BCG stock was thawed and sonicated, then diluted to concentrations of 500 CFU, 1000 CFU, and 1500 CFU, respectively, in culture media. The cells were seeded at 3 x 10^6^ cells per well into a 48-well cell culture plate, and 180 µl of BCG at each concentration was added to different wells and co-cultured for 96 hours. Co-cultures were transferred into 2 mL screw cap tubes and centrifuged at 15,300 xg for 10 minutes. In parallel, 500 µl of sterile water was added to the wells and incubated for at least 5 minutes at room temperature. The supernatant from the screw-cap tubes was carefully removed, and the water from the culture wells added into the corresponding tubes, thoroughly mixed, and transferred to supplemented MGIT tubes. Additionally, 180 µl of BCG at each dilution was added directly to supplemented MGIT tubes, along with 320 µl of supplemented media to reach a total volume of 500 µl. Finally, MGIT tubes were transferred into the BACTEC 960 machine (BD Biosciences, USA) and TTP was recorded.

Inoculum controls for ~500 CFU, ~1000 CFU and ~1500 CFU reached positivity at 7.5 days, 7.2 days and 6.8 days, respectively (**Supplementary Figure S2**). As proof-of-concept, cryopreserved PBMC from a healthy volunteer who had historically received BCG vaccination exhibited distinct TTPs for each inoculum; sample duplicates were consistent (**Supplementary Figure S3**). An inoculum of ~500 CFU was selected going forward as this TTP was most in-line with previously described direct MGIA methods.

#### 2.3.4 MGIA protocol used in this study

Cryopreserved PBMCs were rapidly thawed at 37°C in the water bath to make a cell suspension. The cells were then added to 10 mL of RPMI (10% FBS, 25 mM of HEPES and 2 mM L-glutamine) before being centrifuged at 1500 rpm for 7 minutes. The cells were counted using a Countess™3, Invitrogen™ automated cell counter after 2 hours of incubation at 37°C with 5% CO_2_. PBMCs at a concentration of 3×10^6^ cells/mL were seeded in a 48 well plate at a volume of 300 µl/well together with 180 µl of ~500 CFU of BCG (QSMI) diluted in RPMI and incubated at 37°C with 5% CO_2_ for 96 hours. At the same time, the control tubes for the assay were prepared by the addition of 180 µl of diluted BCG (QSMI) in one MGIT tube together with 800 µl of PANTA antibiotics dissolved into OADC in a sterile manner. Two direct-to-MGIT control tubes were prepared by adding 320 µl of the supplement/tube and placed into the BACTEC 960 machine (BD Biosciences, USA). After the incubation (Day 4), each MGIT tube was supplemented with 800 µl of supplement as described. The co-cultures from the 48 well plates were transferred to 2 mL screw-capped tubes and centrifuged at 15,300 xg for 10 minutes. The detachment and lysis of monocytes from the bottom of the wells was achieved by addition of 500 µl of sterile tissue culture-grade water for 5 minutes. After 5 minutes of incubation, the supernatants were removed from each 2 mL screw-capped tubes, and the water from the corresponding wells were added. The tubes were then pulse vortexed (~1 second) and added to MGIT tubes. The MGIT tubes were inverted to mix thoroughly and placed into the BACTEC 960 machine (BD Biosciences, USA) to record time to positivity (TTP). The conversion of (TTP) read-out into log _10_CFU was achieved by means of a standard curve. BCG net growth in log _10_ CFU was calculated by subtracting the CFU of the inoculum control from the CFU obtained in samples (**Figure 2**).

### 2.4 Statistical analysis

Statistical analysis was conducted using GraphPad Prism (v. 10) software (Boston, MA, USA). Median values for non-parametric data were compared. Comparisons for the groups were conducted using a Kruskal–Wallis test, followed by Dunn’s correction for multiple comparisons. Spearman’s tests were performed for the correlation analyses. Statistical significance was acknowledged for values of p ≤ 0.05.

## 3 Results

### 3.1 Demographics and patient characteristics

A total of 60 subjects (20 ATB patients, 20 LTBI individuals, and 20 HC) were enrolled into the study. The final cohort for immunological analyses comprised 15 ATB, 15 LTBI, and 13 HC after exclusions for low PBMC yield. The clinical and demographic characteristics of the enrolled participants according to their initial group allocation are summarized in **Table 1**. None of the ATB patients or LTBI individuals were receiving anti-TB treatment at the time of sampling. The median (IQR) age of the volunteers in the ATB, LTBI, and HC groups was 61.5 ± 24.5 years, 41 ± 16.25 years, and 34.5 ± 15.75 years, respectively. Most of the participants were female (73.3%). All participants were previously BCG vaccinated.

**Table 1:** Demographic and clinical characteristics of enrolled participants.

Characteristic	ATB (n=20) %	LTBI (n=20) %	HC (n=20) %	Total (n=60)	P-value
Age, years, Median (IQR)	61.50 (47.25-71.75)	41.00 (33.25-49.50)	34.50 (25.25-41.00)		<0.0001
Sex: Female	6 (30)	20 (100)	18 (90)	44 (73.3)	<0.0001
BCG vaccination Status	20 (100)	20 (100)	20 (100)	60 (100)	>0.9999
Past TB History	2 (10)	0	0	2 (3.3)	0.322
Laboratory data					
IGRA, n (%)					0.2213
Positive	NA	1 (5)	3 (15)	4 (7)	
Negative	NA	16 (80)	17 (85)	33 (55)	
Indeterminate	NA	3 (15)	0	3 (5)	
CXR, n (%)					
Normal	2 (10)	20 (100)	20 (100)	42 (73)	<0.0001
Consistent with TB	18 (90)			18 (30)	
Culture, n (%)					
Positive	12 (60)	NA	NA	NA	NA
AFB smear, n (%)					
Positive	11 (55)	NA	NA	NA	NA
PCR, n (%)					
Positive	8 (40)	NA	NA	NA	NA

Data are presented as number (%) unless otherwise described. Demographic characteristics were calculated based on all 20 ATB, 20 LTBI, and 20 HC participants. Kruskal- Wallis test was used to assess the numerical data of the study groups. However, the final experimental analysis included 15 ATB, 15 LTBI, and 13 HC samples. ATB, Active tuberculosis; LTBI, Latent tuberculosis infection; HC, Healthy controls; IQR, Interquartile range; IGRA, Interferon γ release assay; CXR, chest x-ray; AFB, acid fast bacilli; BCG, Bacille Calmette Guerin; PCR, Polymerase chain reaction.

### 3.2 *Ex vivo* mycobacterial growth control is associated with TB disease state

**Figure 3:**
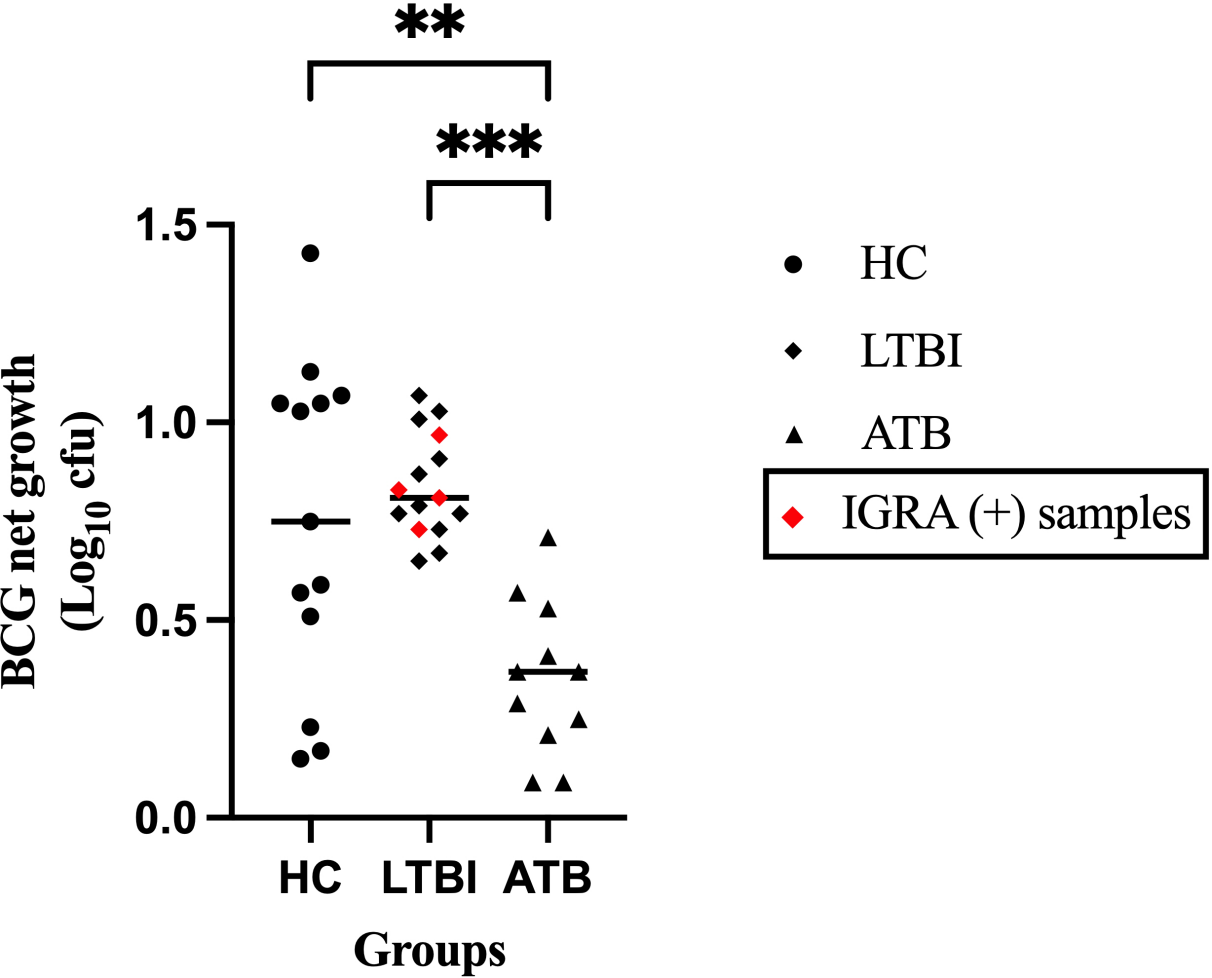
Mycobacterial growth control is associated with TB disease state. Peripheral blood mononuclear cells were co-cultured with ~500 CFU of BCG (QSMI) for 96 hours, and BCG net growth (Log10 CFU) is shown. PBMC samples were collected from healthy volunteers (HC; n=13), latently infected individuals (LTBI; n=15), and active TB disease patients before starting anti-TB treatment (ATB; n=11). Group comparisons were performed using the non-parametric Kruskal-Wallis test, followed by Dunn’s correction for multiple comparisons. A p-value of 0.05 or lower is considered statistically significant **p < 0.01 and ***p < 0.001. 4 samples from the ATB group were contaminated and excluded from the study..

To evaluate mycobacterial growth inhibition in PBMC from participants with different TB disease states, the net growth of BCG was quantified following the direct MGIA and expressed as Log_10_ colony-forming units (CFU). The ATB group showed significantly greater control of mycobacterial growth compared with the LTBI and HC groups (p = 0.0005 and p = 0.0127, respectively), with a heterogenous distribution of growth control among the HC group (**Figure 3**). There was no difference in growth control between IGRA (+) and IGRA (-) LTBI participants.

### 3.3 NK cell frequency differs between TB disease states

**Figure 4:**
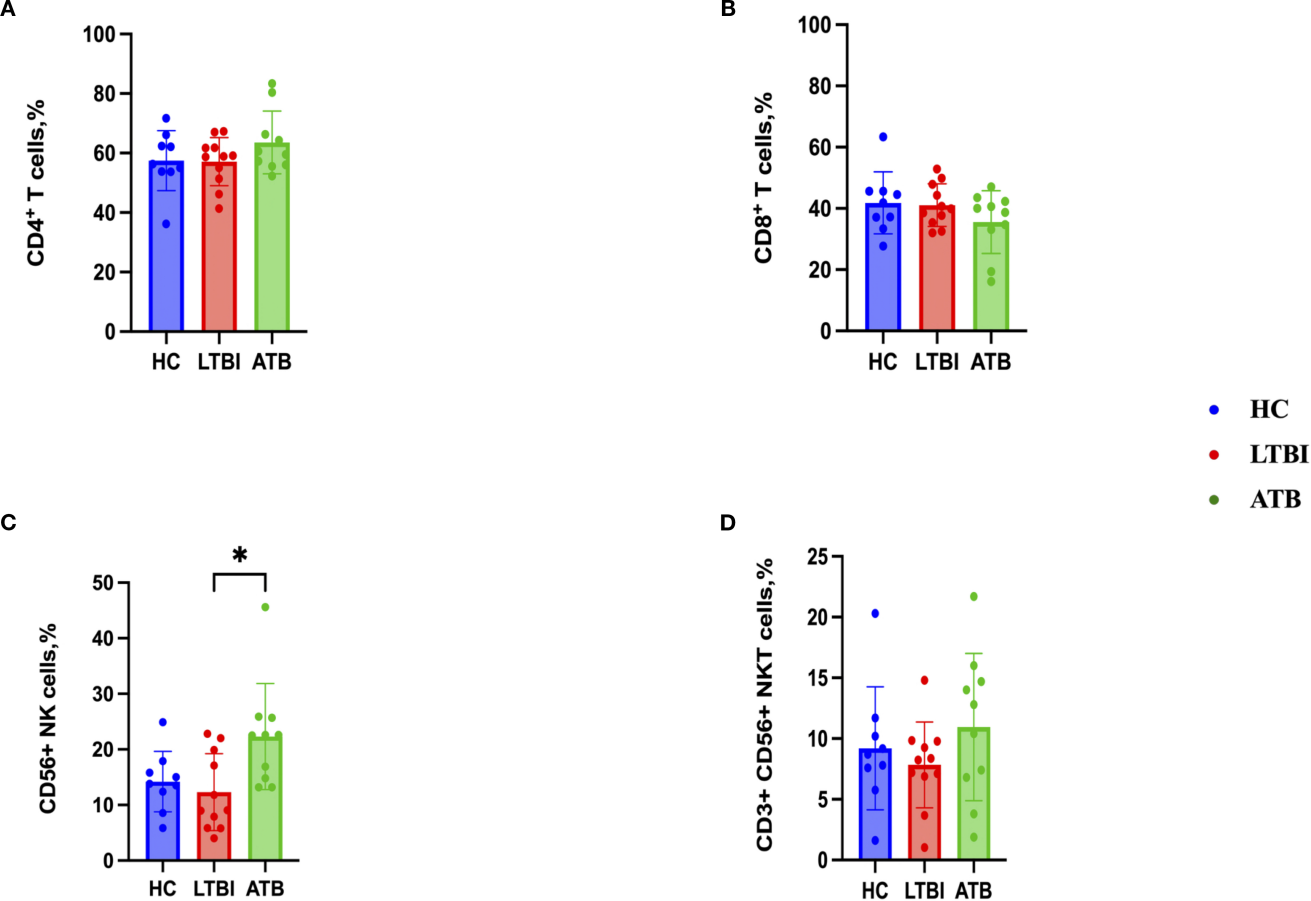
Frequencies of immune cell populations in peripheral blood mononuclear cells isolated from each of three participant groups HC, n=11; LTBI, n=8; ATB, n=10; after PPD stimulation. Percentage of CD4+ T cells **(A)**, CD8+ T cells **(B)**, CD56+ NK cells **(C)**, CD3+ CD56+ NKT cells **(D)**. Graphs are box and whisker plots with median ± interquartile range (IQR) with additional dot plot representing individual donors. The statistical significance of difference was performed by using Kruskal-Wallis followed by Dunn’s correction for multiple comparisons. A p-value of 0.05 or lower is considered statistically significant, *p< 0.05.

To characterize the CD4+ and CD8+ T cell, CD56+ NK cell and CD3+ CD56+ NKT cell responses, flow cytometry analysis was performed after stimulation with PPD of the PBMCs from 10 ATB, 11 HC and 8 LTBI participants for which sufficient cell numbers were available. The gating strategies for the cytokine expressions of CD4+ and CD8+ T cells, CD3- CD56+ NK cells and CD3+ CD56+ NKT cells are shown in (**Supplementary Figures S5–S10**) respectively. There were no statistically significant differences between groups in the percentages of CD4+ T cells, CD8+ T cells or NKT cells (**Figures 4A, B, D**). However, the ATB group had a significantly higher frequency of NK cells compared to the LTBI group (p< 0.05) (**Figures 4A–D**).

### 3.4 Modest differences in proinflammatory cytokine production between TB disease states

**Figure 5:**
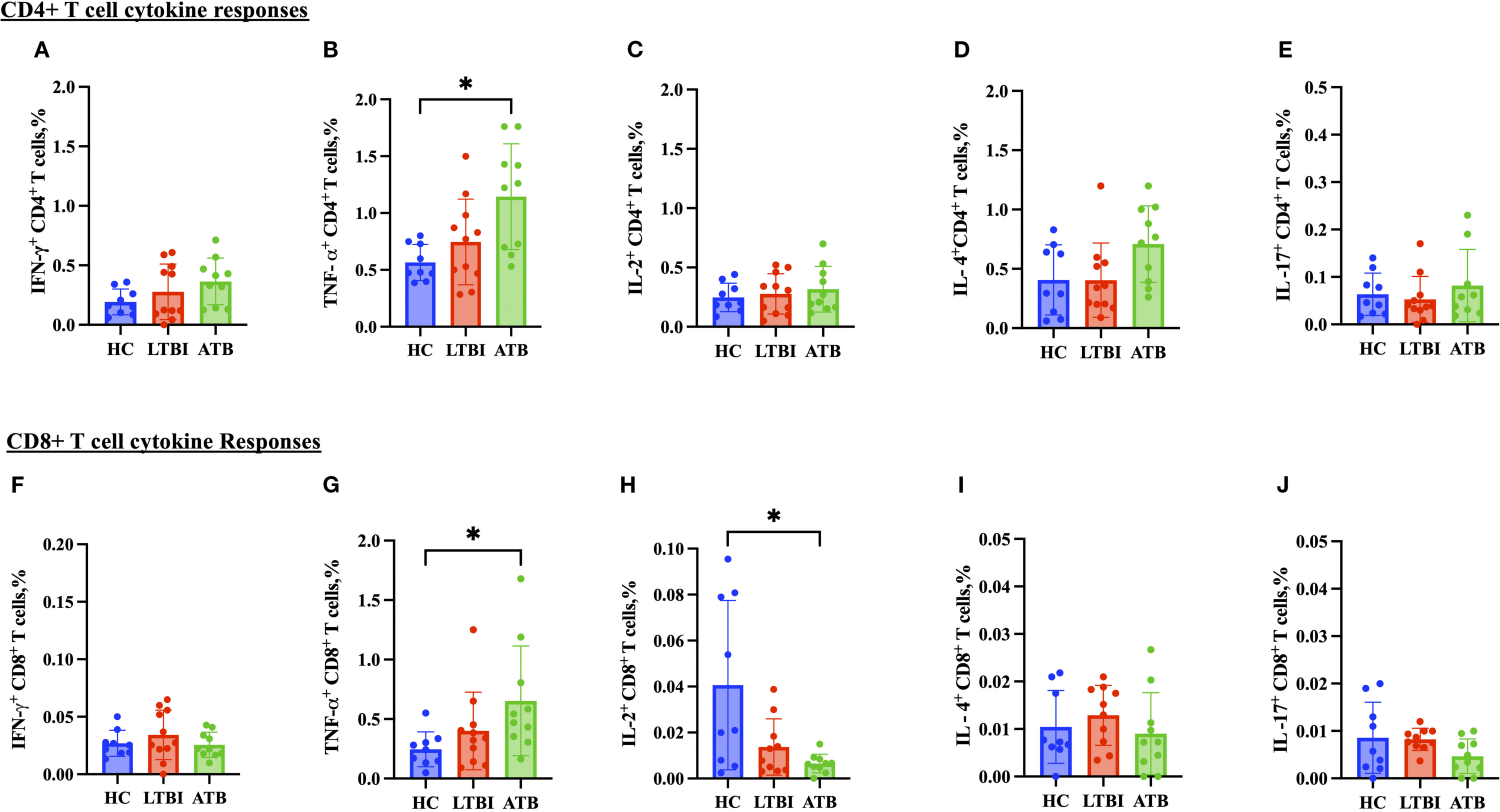
Frequencies of CD4+ and CD8+ T cell cytokines in PBMCs isolated from each of three participant groups HC, n=11; LTBI, n=8; ATB, n=10; after PPD stimulation. Percentage of IFN-γ+ CD4+ T cells **(A)**, TNF-α+ CD4+ T cells **(B)**, IL-2+ CD4+ T cells **(C)**, IL-4+ C D4+ T cells **(D)**, IL-17+ CD4+ T cells **(E)**, IFN-γ+ CD8+ T cells **(F)**, TNF-α+ CD8+ T cells **(G)**, IL-2+ CD8+ T cells **(H)**, IL-4+ CD8+ T cells **(I)** and IL-17+ CD4+ T cells **(J)**. Graphs are box and whisker plots with median ± interquartile range (IQR) with additional dot plot representing individual donors. The statistical significance of difference was performed by using Kruskal-Wallis followed by Dunn’s correction for multiple comparisons. A p-value of 0.05 or lower is considered statistically significant, *p< 0.05.

**Figure 6:**
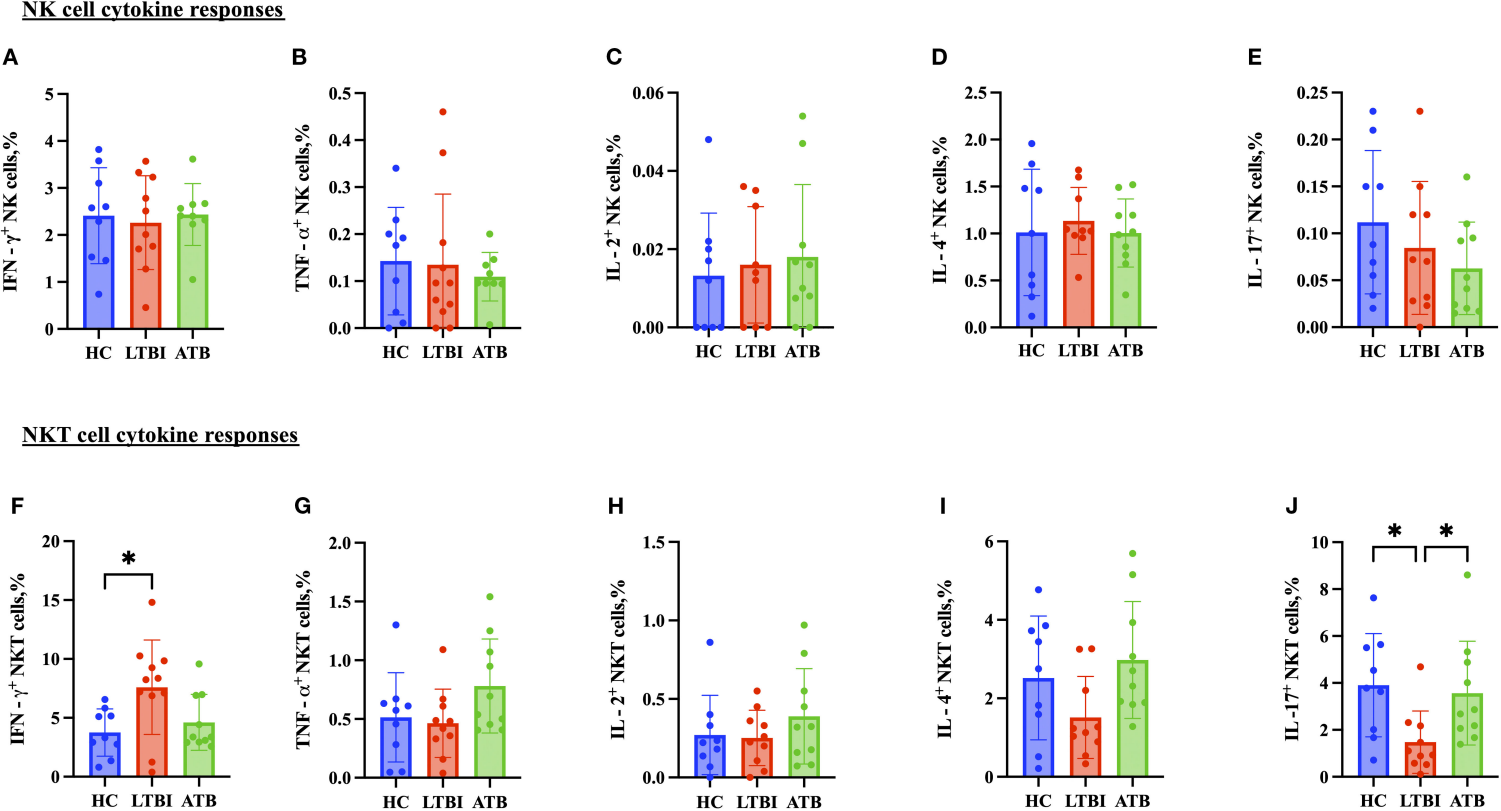
Frequencies of CD56+ NK cell and CD3+ CD56+ NKT cell cytokines in PBMCs isolated from each of three participant groups HC, n=11; LTBI, n=8; ATB, n=10; after PPD stimulation. Percentage of IFN-γ+ CD56+ NK cells **(A)**, TNF-α+ CD56+ NK cells **(B)**, IL-2+ CD56+ NK cells **(C)**, IL-4+ CD 56+ NK cells **(D)**, and IL-17+ CD56+ NK cells (E) IFN-γ+ CD3+CD56+ NKT cells **(F)**, TNF-α+ CD3+CD56+ NKT cells **(G)**, IL-2+ CD3+CD56+ NKT cells **(H)**, IL-4+ CD3+CD56+ NKT cells **(I)**, and IL-17+ CD3+CD56+ NKT cells **(J)**. Graphs are box and whisker plots with median ± IQR; with additional dot plot representing individual donors. the statistical significance of difference was performed by using Kruskal Wallis followed by Dunn’s multiple comparisons test. A p-value of 0.05 or lower is considered statistically significant, *p < 0.05.

CD4+ T cell TNF-α production was significantly higher in the ATB group (**Figure 5B**) compared to the HC group, whereas IFN-γ, IL-2, IL-17 and IL-4 responses were comparable (**Figures 5A, C–E**). Regarding the cytokines produced by CD8+ T cells, there was a significantly lower IL-2 response in the ATB group (p < 0.05) (**Figure 5H**) compared with HC. TNF-α response was higher in the ATB group (p < 0.05) (**Figure 5G**) compared with HC, but other cytokine responses were comparable (**Figures 5F, I, J**). There were no significant differences between groups in NK cell cytokine production (**Figure 6**), but for NKT cells the IFN-γ level was significantly higher in the LTBI group compared with HCs (p < 0.05) (**Figure 6F**). The IL-17 response was significantly higher in the HC and ATB groups compared to the LTBI group (p < 0.05) (**Figure 6J**). No significant differences of other cytokines between groups in NKT cells (**Figure 6G–I**).

### 3.5 Control of mycobacterial growth is associated with CD4+ T cell IL-4 responses and CD4+ and CD8+ T cell TNF-α responses

**Figure 7:**
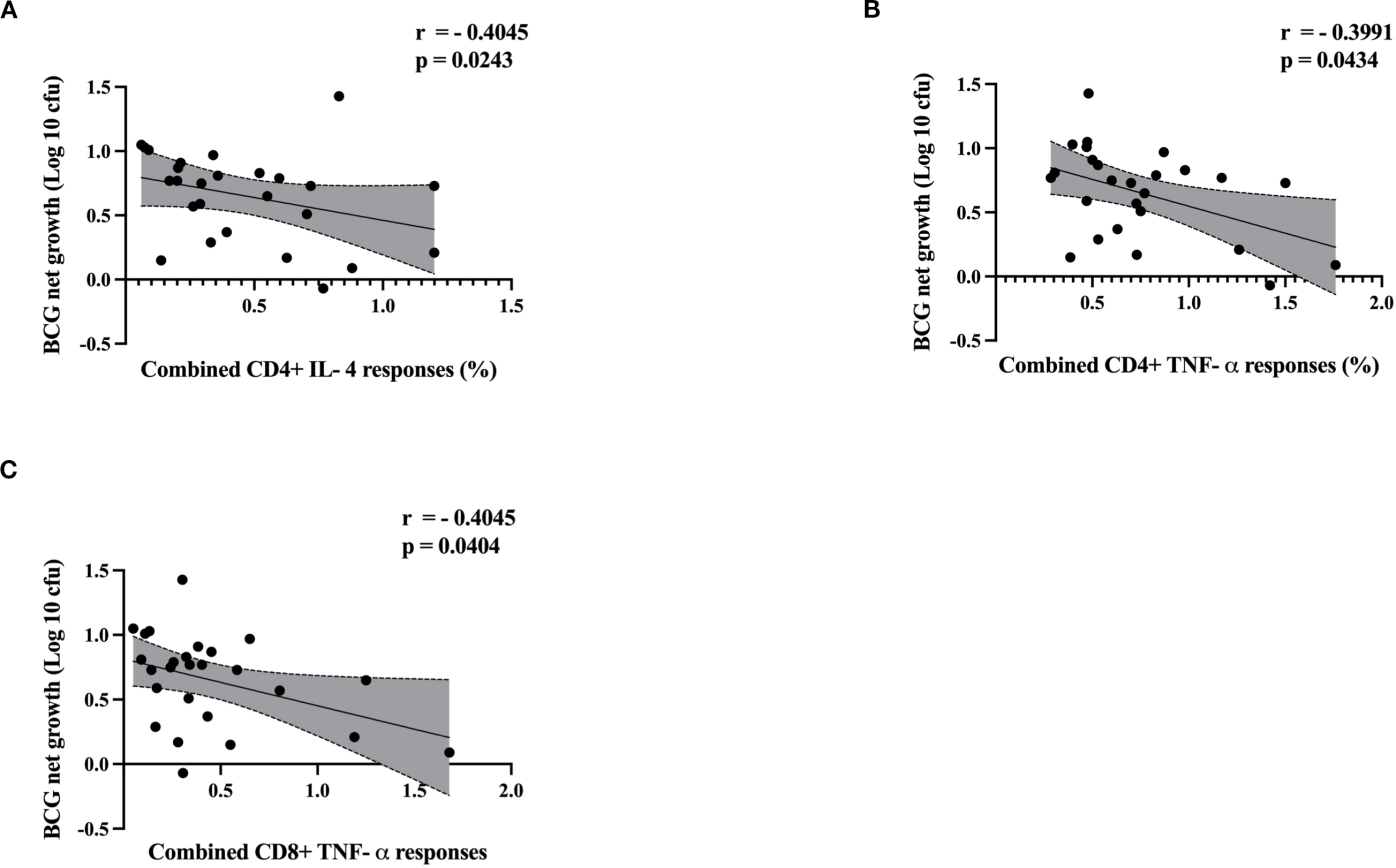
Scatter plots illustrate the correlation between cytokine responses and BCG net growth (log 10 cfu) by MGIA. ATB (n = 7), LTBI (n=11) and HC (n=8). Correlation between BCG net growth (log 10 cfu) and **(A)** CD4+ IL-4, **(B)** CD4+ TNF-α and **(C)** CD8+ TNF-α. Data were analyzed using Spearman’s correlation, and p-values were calculated. Each point represents a single value, and shaded regions represent 95% confident intervals of regression line. A p-value of 0.05 or lower is considered statistically significant.

Next, we analyzed the correlation between control of mycobacterial growth in the MGIA and cytokine responses by flow cytometry using matched samples where available (ATB; n= 7, LTBI; n=11 and HC; n= 8). Group-specific correlation analyses are presented in **Supplementary Figures S13** through **Supplementary Figure S15**. There was a significant inverse correlation between CD4+ T cell IL-4 responses and BCG net growth (rho = -0.4045, p = 0.0243) (**Figure 7A**), and between CD4+ T cell TNF-α responses and BCG net growth (rho = -0.3991,p = 0.0434) (**Figure 7B**). There was also a significant inverse correlation between CD8+ T cell TNF-α responses and BCG net growth (rho = 0.4045, p = 0.0404) (**Figure 7C**).

## 4 Discussion

Despite widespread BCG vaccination and decades of research, tuberculosis continues to be a leading infectious cause of death globally. A significant challenge in TB control lies in the absence of effective tools for assessing protective immunity and distinguishing between stages of infection. In this study, we focused on characterizing the immune responses and functional ability of PBMCs to inhibit mycobacterial growth across individuals with active TB disease, latent TB infection, and healthy controls in Southern Thailand. By integrating the MGIA with flow cytometric analysis of immune phenotypes and cytokine profiles, we provide novel insights into host immune functionality in TB in this population. Implementing the MGIA technique in a new laboratory conducting vaccine development research can reduce costs and expedite preclinical development, as the assay requires only a blood sample to determine functional growth control and immune responses and can utilize either whole blood or PBMCs (20, 22).

In this study, PBMCs from ATB patients exhibited superior control of mycobacterial growth compared to the HC and LTBI groups. During active TB disease, the immune system undergoes chronic activation, characterized by elevated levels of pro-inflammatory cytokines and effector cells circulating in the peripheral blood. This activation may associate with antigen load and confer superior *ex vivo* mycobacterial control. Another possible explanation is that PBMCs represent circulating immunity, whereas TB primarily manifests in lung-resident cells. Enhanced peripheral control in ATB may indicate systemic immune mobilization, while local lung exhaustion or suppression facilitates disease progression. Alternatively, ongoing *M.tb* infection may epigenetically reprogram monocytes/macrophages, making them more responsive to secondary stimulation *ex vivo* (23).

Consistent with our findings, reduced mycobacterial growth has previously been observed in ATB patients compared to healthy controls in a UK population (24). Interestingly, that study was conducted using whole blood, which encompasses both innate and adaptive immune mechanisms, while we used PBMCs. Joosten et al. further indicated that robust control of mycobacterial outgrowth was observed in a subgroup of individuals with recent exposure to *M.tb*, while it was not observed in individuals with long-term LTBI (11). More recently, a 2025 pre-print reported that PBMCs from ATB patients conferred superior *M. tb* growth control, attributing it to activated extracellular killing mechanisms such as cytokine release in supernatants (25). A study conducted in preclinical animal models (rhesus and cynomolgus macaques) demonstrated a substantial reduction in mycobacterial growth following incubation with cells derived from post-*M. tb* infection in comparison to cells obtained from the same animals prior to infection (26). Cheon et al. established a whole-blood bactericidal assay where intracellular mycobacterial survival varied by virulence and decreased ~0.3 log after repeated BCG vaccination. This aligns with our study, where vaccination/exposure enhanced MGIA growth inhibition, reinforcing the assay’s potential utility as a functional mycobacterial immunity surrogate (27).

However, outcomes have been less consistent in studies in South Africa and South Korea, suggesting the effect may be population-dependent (28, 29). These studies show inverse patterns, with LTBI or vaccinated individuals exhibiting enhanced mycobacterial control than those with ATB, aligning with the “expected” protective paradigm. Discrepancies may arise from differences in cohort, MGIA mycobacterial strain, or quantification endpoints.

We observed higher percentages of NK cells in ATB patients compared with the LTBI group, suggesting they may play a role in the early immune response, for example through antibody-dependent cell-mediated cytotoxicity (ADCC). However, previous studies on NK cells in TB have reported mixed results. Yoneda et al. found significantly higher NK cell activity in ATB patients compared to HC (30), while Zhou et al. observed a similar, albeit non-significant trend (31, 32). However, Garand et al. found no difference in NK cell frequencies between pre-treatment and post-treatment TB household contact groups (33), and some studies have even reported lower NK cell levels in ATB patients than in individuals with LTBI (31, 34–36). These inconsistencies may reflect differences in study populations, infection stages, or levels of immune activation.

We also observed elevated levels of TNF-α-producing CD4^+^ T cells in patients with ATB disease compared to those with LTBI, aligning with prior research that supports TNF-α as a potential biomarker to distinguish ATB from LTBI (37–39). TNF-α, primarily produced by macrophages and monocytes, is a pro-inflammatory cytokine crucial for inducing cell necrosis, apoptosis, and resistance to infections and cancers (40, 41). The elevated TNF-α levels in the ATB group may contribute not only to immune defense but also to lung tissue damage, including necrosis and cavitation. However, a study by Wu et al. reported higher levels of IL-2, TNF-α, and IFN-γ in LTBI individuals upon PPD stimulation, contrasting with our findings (42). These contrasting observations underscore the complex role of CD4+ T cell cytokines, particularly TNF-α, in TB pathogenesis and potential diagnostic differentiation.

ATB patients also exhibited higher frequencies of CD8+ TNF-α-producing T cells compared to HC and individuals with LTBI, consistent with previous studies (43). CD8+ T cells may play an important role in controlling *M.tb* infection, particularly intracellularised bacteria, through classical MHC class 1-restricted mechanisms (44–47). Although initially considered less critical than CD4+ T cells, CD8+ T cells are now recognized as key contributors to host defense, capable of killing infected cells via cytotoxic molecules such as perforin, granzymes, and granulysin (45, 46, 48, 49). In addition, NKT cells are known to produce high levels of IL-17 while secreting low levels of interferon IFN-γ (50–52). Their ability to recognize microbial lipid antigens, particularly from gram-negative bacteria, and to produce a broad range of cytokines may contribute to the increased IL-17 levels observed in ATB patients this study (53–55).

Interestingly, control of mycobacterial growth correlated with CD4+ TNF-α, CD8+ TNF-α and CD4+ IL-4 responses. TNF-α is a pro-inflammatory cytokine and its pivotal role has been demonstrated in murine models, where deficiency in (TNFRp55) gene and TNF, or Lymphotoxin-alpha (LT-α) leads to fatal mycobacterial infection (56, 57). In humans, several studies reported that anti-TNF therapies have been shown to compromise host defense against TB and reactivate latent infection (58–61). Importantly, none of the participants in this study had known autoimmune diseases or were receiving immunosuppressive treatment. IL-4, a Th2 cytokine, promotes humoral immunity but can suppress the protective Th1 response in TB (62–64). It also induces alternative macrophage activation, which may support mycobacterial survival. A shift from Th1 to Th2 dominance during TB progression has been reported, disrupting Th1/Th2 balance and facilitating the progression of TB disease (65–67). The inverse correlation of IL-4 with MGIA growth challenges its proposed detrimental role as a Th2 cytokine; while IL-4 may regulate inflammation or synergize with cytokines for containment, elevated IL-4 in ATB has been associated with exacerbated pathology. Further investigations such as IL-4 neutralization in MGIA or Th1/Th2 co-cultures, could clarify mechanisms in larger cohorts. Although CD8+ T cells are primarily cytotoxic, they can also produce IL-17, which contributes to neutrophil recruitment and granuloma formation (45, 46, 67). Overall, these findings reinforce the important role of TNF-α-producing CD4+ and CD8+ T cells in the immune control of mycobacterial infections and the complexity of host-pathogen interactions. As none of the participants were receiving therapy at the time of sampling, our findings indicate that the observed cytokine responses are a consequence of physiological immune activity rather than drug-induced modulation.

The current study presents certain limitations. LTBI classification in our study was based on established inclusion criteria emphasizing high-risk *M.tb* exposure and TST positivity (≥10 mm induration), in line with WHO recommendations for LTBI diagnosis in high-burden settings where no gold standard exists. It is possible that this group included uninfected individuals, particularly those who were concurrently IGRA negative, thus potentially diluting group differences. However, we feel that this reflects real-world heterogeneity and we were able to nonetheless identify significant differences between groups. The study’s modest final sample sizes (ATB n=15, LTBI n=15, HC n=13) reflect logistical constraints in high-burden settings, including limited PBMC recovery post-thawing, high assay costs, and ethical challenges in recruiting ATB patients, limiting statistical power for detecting subtle effects and potentially underrepresenting intragroup variability, reducing generalizability to broader populations. While we observed increased NK cell frequency and IL-17 production by NKT cells in ATB patients, the lack of broader functional assays and deeper phenotypic resolution limits our ability to fully define their contributions. Future studies incorporating detailed functional analyses such as cytotoxic capacity, cytokine production profiling, and subset-specific phenotyping will be necessary to clarify the precise roles of NK and NKT cells in TB immunopathogenesis. Furthermore, combining data from ATB, LTBI, and HC groups for correlation analyses may obscure group-specific differences. Cytokine measurements were conducted *in vitro* and may not fully capture the dynamic and intricate immune responses that occur *in vivo*. Additionally, the MGIA exhibits inherent variability due to factors such as precise bacterial strain, culture conditions, incubation duration, and growth measurement techniques. Another limitation of using BCG as the MGIA inoculum is its attenuated nature. This strain lacks specific virulence factors in the RD1 region, which may under-represent immune mechanisms targeting *M.tb*-specific antigens and reduce its biological relevance. However, BCG and *M.tb* have a very high degree of genomic homology, and BCG provides a practical surrogate for assessing mycobacterial growth restriction that is more feasible from a logistical and biosafety perspective, particularly in low-resource settings, maximising transferability and impact of the assay. Furthermore, several MGIA studies have directly compared using *M.tb* and BCG and demonstrated that TTP and growth inhibition between the two strains are strongly correlated (7, 24, 29, 68).

Although our cross-sectional design captures the immune response at a moment in time, it precludes temporal insights. Longitudinal studies could elucidate dynamics, such as TNF-α/MGIA shifts post-treatment or predictors of LTBI-to-ATB progression. Future work in this cohort will prioritize such designs to validate correlates. Future studies involving larger, well-defined cohorts and additional functional assays are necessary to further elucidate the correlation between cytokine responses and MGIA outcomes. Investigating the mechanisms by which cytokines influence mycobacterial control and targeting specific cytokines for therapeutic intervention may also provide novel insights. Furthermore, exploring cytokine dynamics across various stages of TB and LTBI individuals could enhance our comprehension of protective versus pathogenic immune responses. TNF-α–producing CD4+ and CD8+ T cells correlated with improved mycobacterial control, suggesting they may be more reliable correlates of protection than IFN-γ alone. This highlights the importance of inducing TNF-α–driven multifunctional T cell responses in TB vaccine design. Our findings demonstrating positive correlation between mycobacterial growth control and CD4+ TNF-α, CD8+ TNF-α, and CD4+ IL-4 responses emphasize the significance of vaccine candidates that induce polyfunctional T cell responses. These cytokine profiles may serve as potential immune correlates of protection. Notably, TNF-α-mediated responses from both CD4+ and CD8+ T cells likely reflect a robust pro-inflammatory milieu essential for mycobacterial containment. Future vaccine strategies may benefit from targeting the induction of these cytokine responses, both as functional endpoints and as surrogate markers of protective efficacy beyond IFN-γ alone. Integrating these cytokine correlates into preclinical and clinical assessments may advance the discovery of more effective TB vaccines.

## 5 Conclusion

This study contributes to the elucidation of the complex immunological mechanisms underlying the control of *M.tb* and validates the pivotal role of TNF-α-producing CD4+ and CD8+ T cells, as well as IL-4 responses, in orchestrating host immunity – specifically in a population from Southern Thailand. By synergistically integrating MGIA with flow cytometric profiling, we establish a correlation between cytokine responses and mycobacterial growth inhibition, thereby providing potential insights into functional immune biomarkers.

## Data Availability

The raw data supporting the conclusions of this article will be made available by the authors, without undue reservation.
