# Supplementary material for: Functional and phenotypic characterization of peripheral blood mononuclear cells from tuberculosis patients in Southern Thailand
Source: Front Immunol. 2025 Oct 7;16:1639808. doi: 10.3389/fimmu.2025.1639808 (PMC12537796; doi:10.3389/fimmu.2025.1639808)
Supplement: Supplementary file 1 [file SupplementaryFile1.docx]

**Supplementary Material**

**
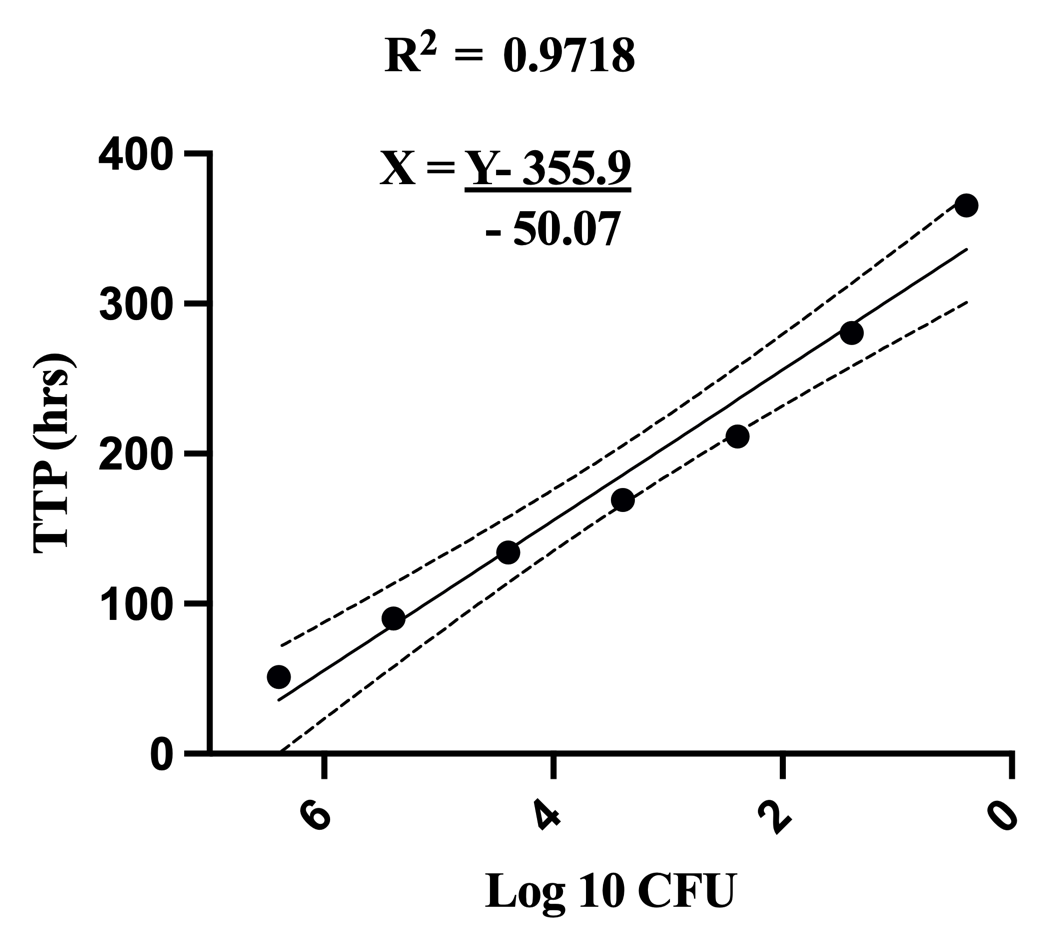
**

**Figure S1. In-house BCG standard curve.** The representative graph shows a standard curve plotting time to positivity (TTP) from the BACTEC MGIT system against log 10-transformed colony-forming units (CFU). The statistical analysis was performed using simple linear regression on a semi-log scale.


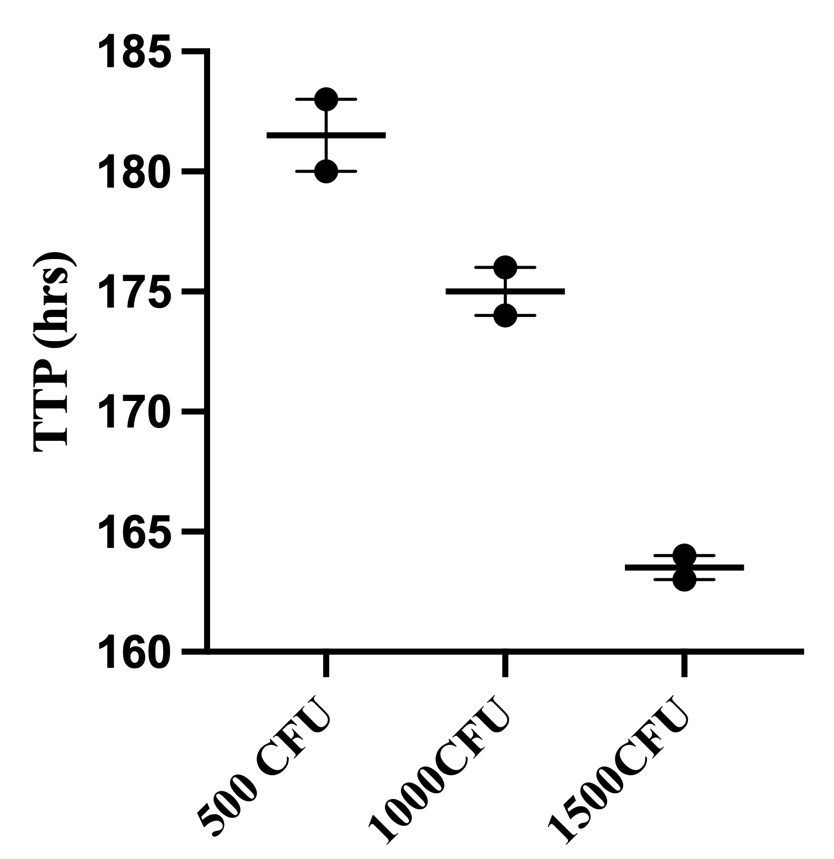


**Figure S2. The BCG inoculum in different concentration.** The time to positivity (TTP) of the inoculum control at different concentrations of BCG. Each inoculum was performed in duplicate with the mean and standard error of mean (SEM) shown.


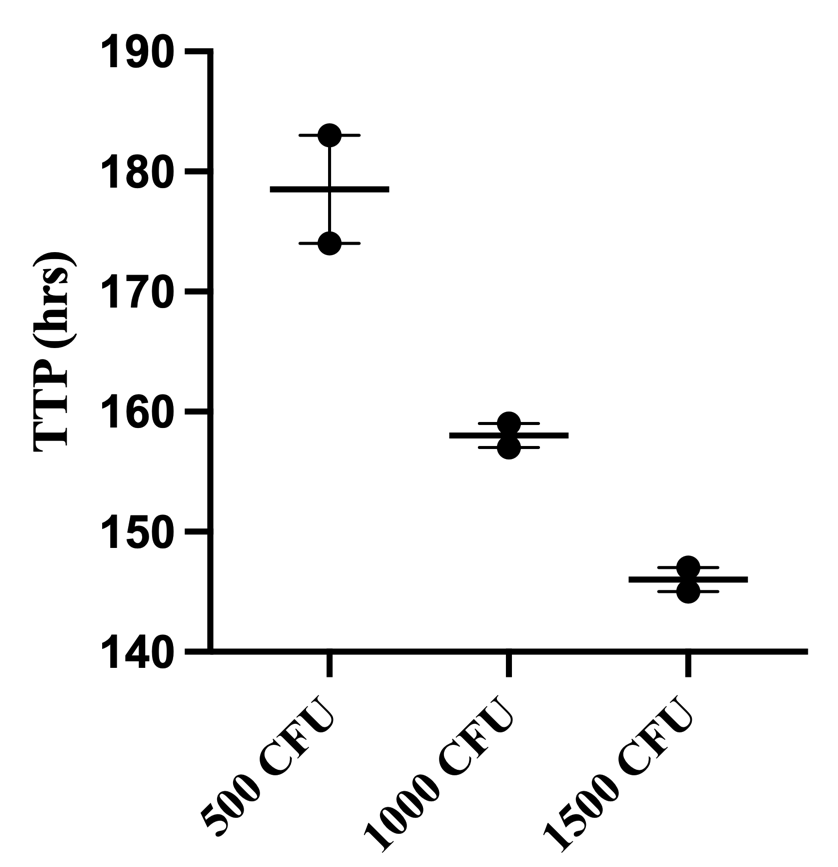


**Figure S.3. The growth control of PBMCs at various inoculum levels.** PBMCs were obtained from a BCG-vaccinated healthy volunteer. Different inoculum levels (500 CFU, 1000 CFU, and 1500 CFU of BCG) were added to the cultured assay. Each inoculum condition was tested in duplicate, with each data point representing the mean and standard error of mean (SEM) of the duplicates.


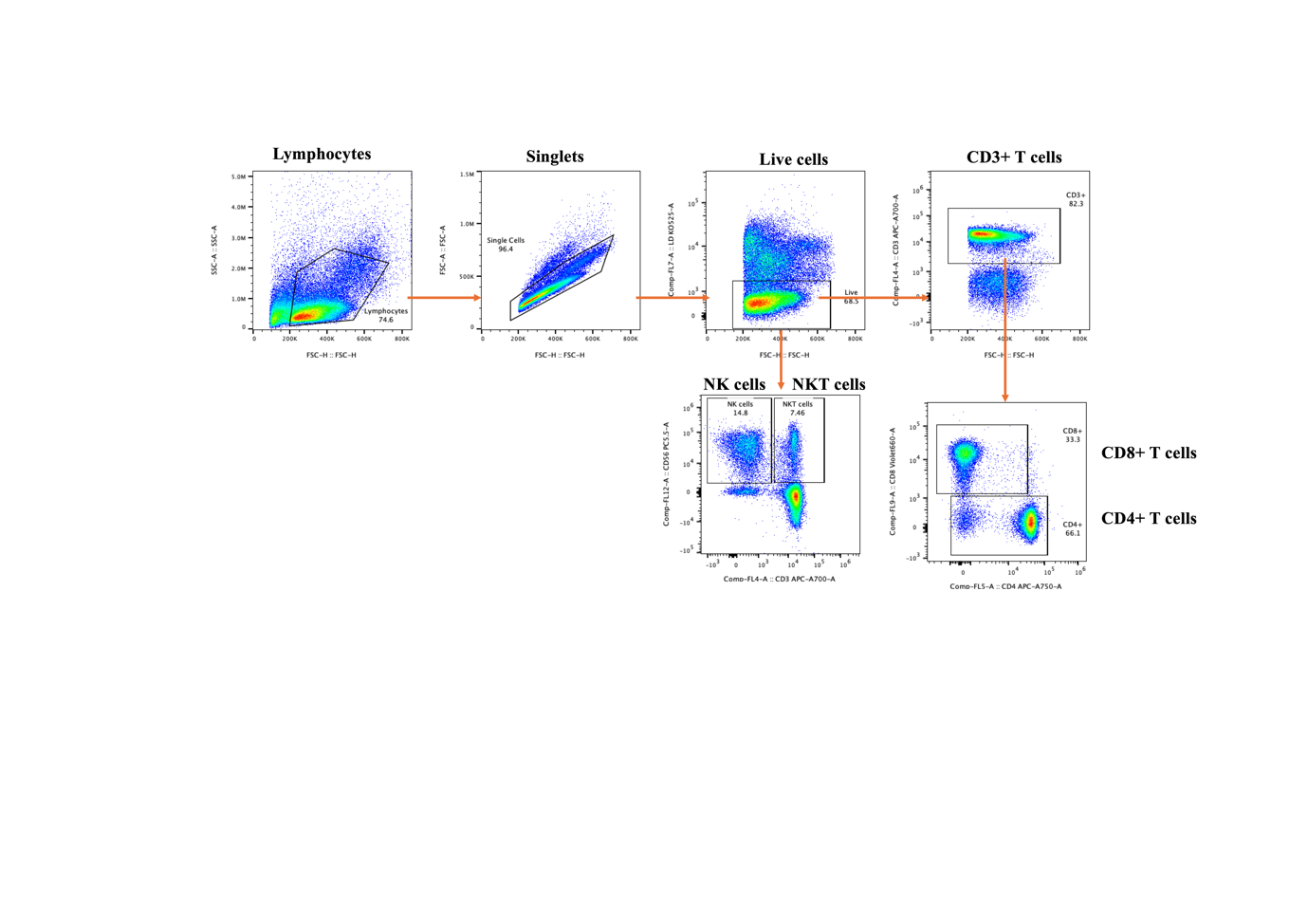


**Figure S.4.Representative flow cytometry plots of T cells, NK and NKT cell populations in PBMCs *of* ATB group.** Lymphocytes were first gated based on forward scatter (FSC) and side scatter (SSC) properties. Singlet cells were selected to exclude doublets and debris. Live cells were identified using a viability dye. CD3+ T cells were gated to distinguish T cell subsets. Among the CD3+ population, CD4+ and CD8+ T cells were identified. Additionally, NK cells (CD3- CD56+) and NKT cells (CD3+ CD56+) were gated to analyze their frequencies within the lymphocyte population. Percentages of each cell population are indicated in the respective gated regions. Data were analyzed using the FlowJo software (Tree Star, Ashland, OR, USA).


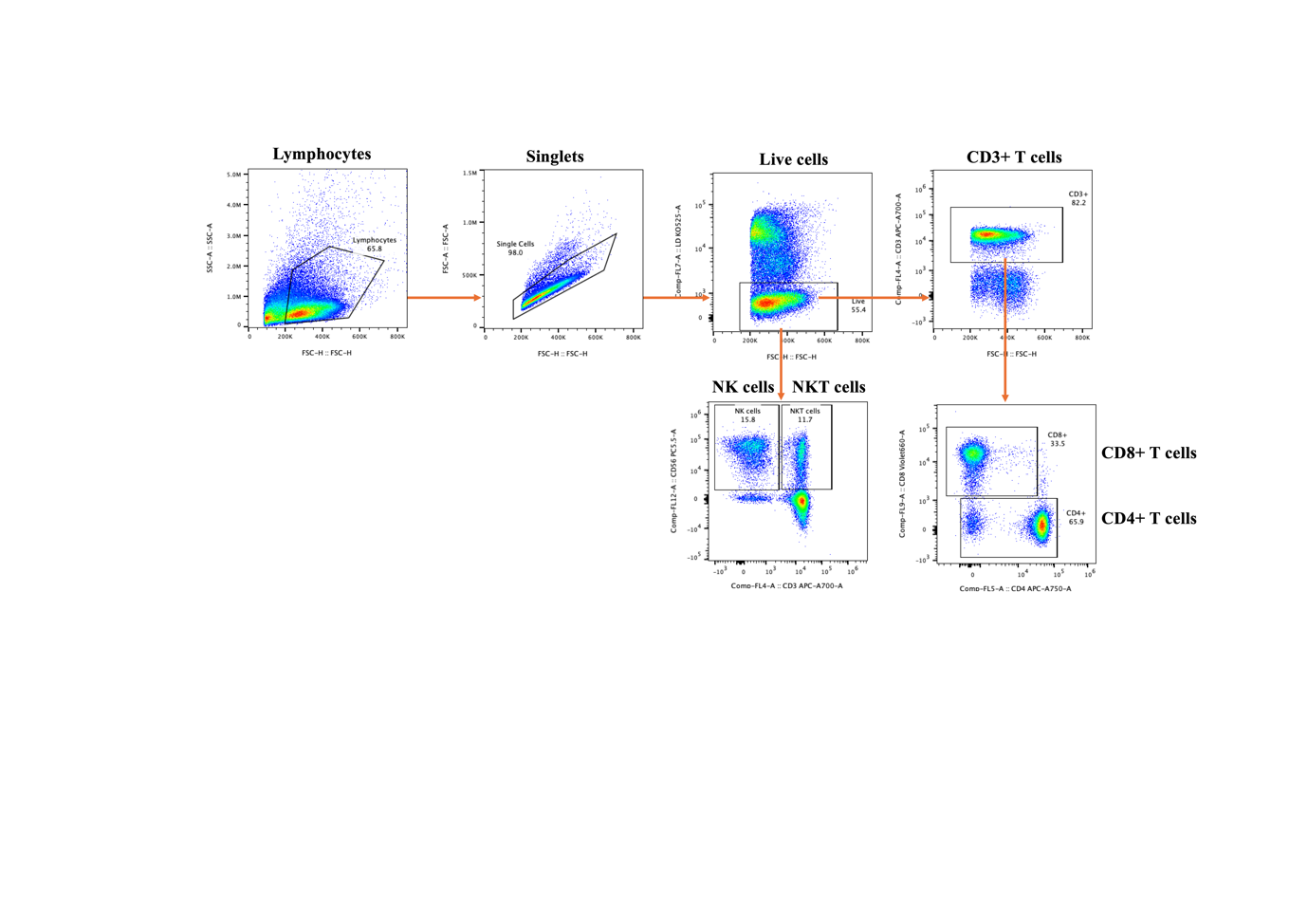


**Figure S.5. Figure S.4.Representative flow cytometry plots of T cells, NK and NKT cell populations in PBMCs of HC group.** Lymphocytes were first gated based on forward scatter (FSC) and side scatter (SSC) properties. Singlet cells were selected to exclude doublets and debris. Live cells were identified using a viability dye. CD3+ T cells were gated to distinguish T cell subsets. Among the CD3+ population, CD4+ and CD8+ T cells were identified. Additionally, NK cells (CD3- CD56+) and NKT cells (CD3+ CD56+) were gated to analyze their frequencies within the lymphocyte population. Percentages of each cell population are indicated in the respective gated regions. Data were analyzed using the FlowJo software (Tree Star, Ashland, OR, USA).


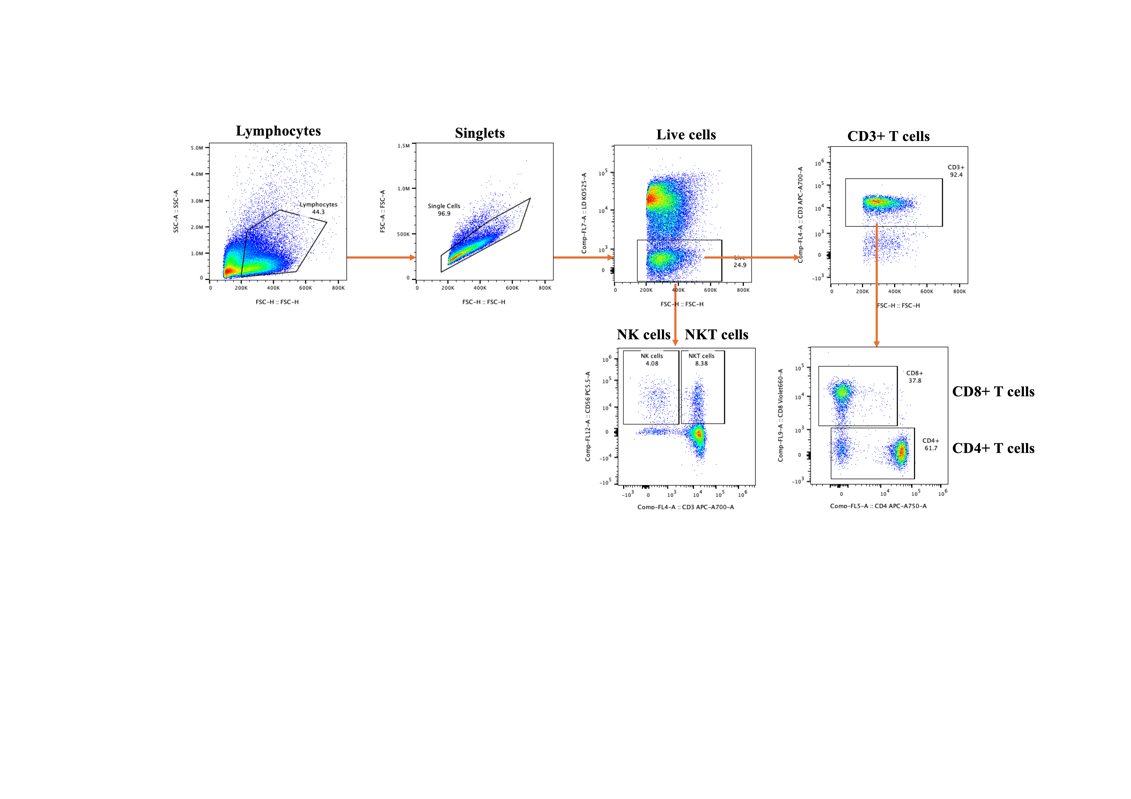


**Figure S.6. Representative flow cytometry plots of T cells, NK and NKT cell populations in PBMCs of LTBI group.** Lymphocytes were first gated based on forward scatter (FSC) and side scatter (SSC) properties. Singlet cells were selected to exclude doublets and debris. Live cells were identified using a viability dye. CD3+ T cells were gated to distinguish T cell subsets. Among the CD3+ population, CD4+ and CD8+ T cells were identified. Additionally, NK cells (CD3- CD56+) and NKT cells (CD3+ CD56+) were gated to analyze their frequencies within the lymphocyte population. Percentages of each cell population are indicated in the respective gated regions. Data were analyzed using the FlowJo software (Tree Star, Ashland, OR, USA).


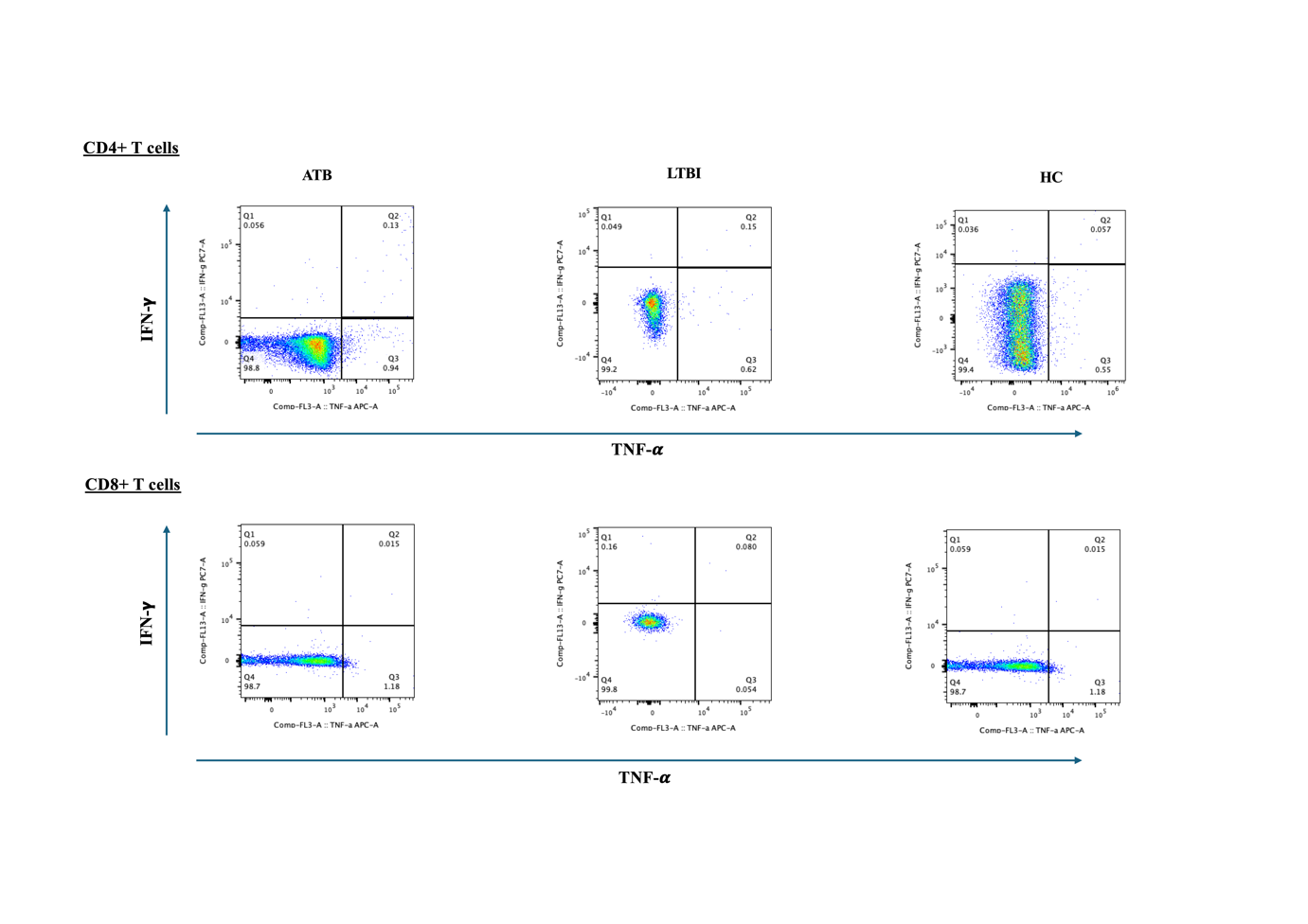


**Figure S.7. Representative flow cytometry plots showing IFN-𝛄 and TNF-𝜶 production in CD4+ and CD8+ T cells among ATB, LTBI and HC groups.** The top row represents NK cells, white the bottom row represents NKT cells. IFN-𝛄 expression is shown on the y-axis and TNF-𝜶 expression is shown on the x-axis. Quadrants indicate different cytokine-producing populations. Q1(IFN-𝛄 + TNF-𝜶-), Q2 (IFN-𝛄 + TNF-𝜶 +), Q3 (IFN-𝛄 - TNF-𝜶 +) and Q4 (double negative). Data illustrate differences in cytokine production across the groups.

**
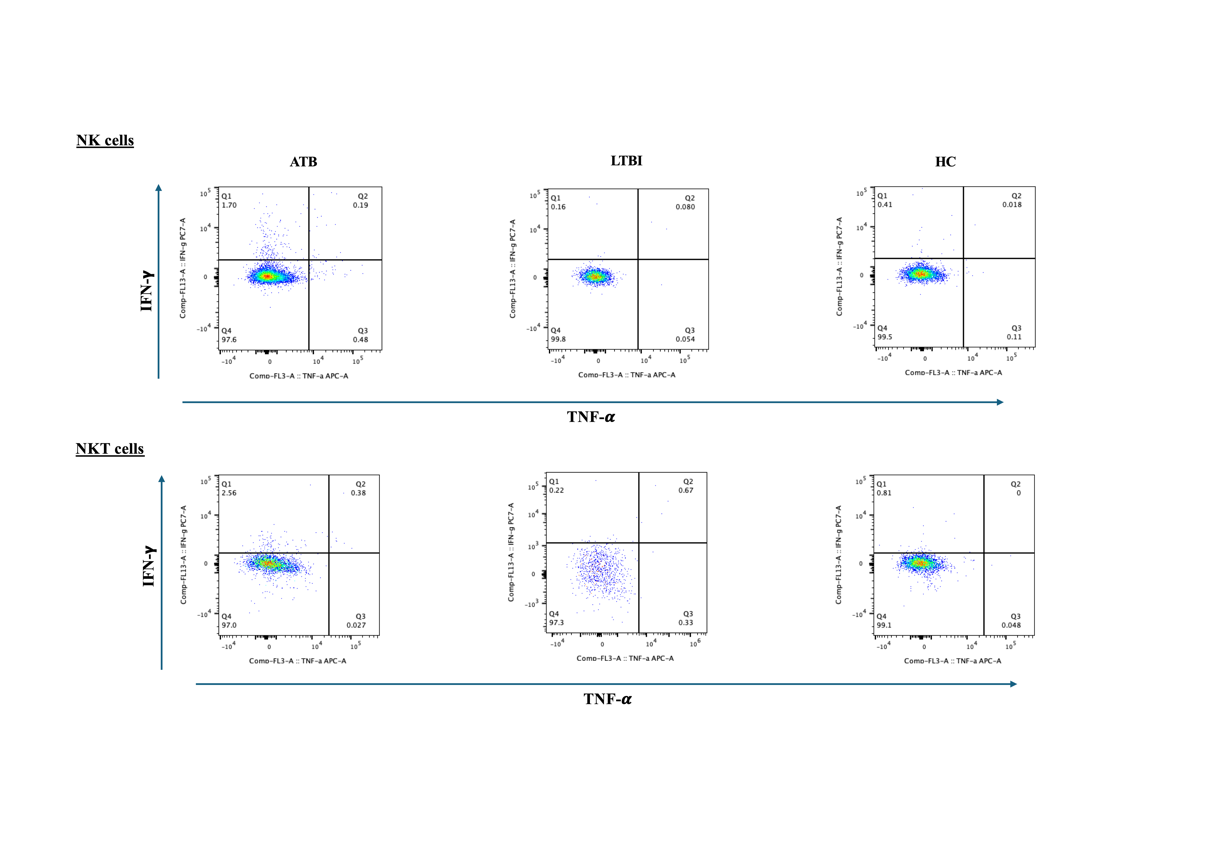
**

**Figure S.8. Representative flow cytometry plots showing IFN-𝛄 and TNF-𝜶 production in NK and NKT cells among ATB, LTBI and HC groups.** The top row represents NK cells, white the bottom row represents NKT cells. IFN-𝛄 expression is shown on the y-axis and TNF-𝜶 expression is shown on the x-axis. Quadrants indicate different cytokine-producing populations. Q1(IFN-𝛄 + TNF-𝜶-), Q2 (IFN-𝛄 + TNF-𝜶 +), Q3 (IFN-𝛄 - TNF-𝜶 +) and Q4 (double negative). Data illustrate differences in cytokine production across the groups.


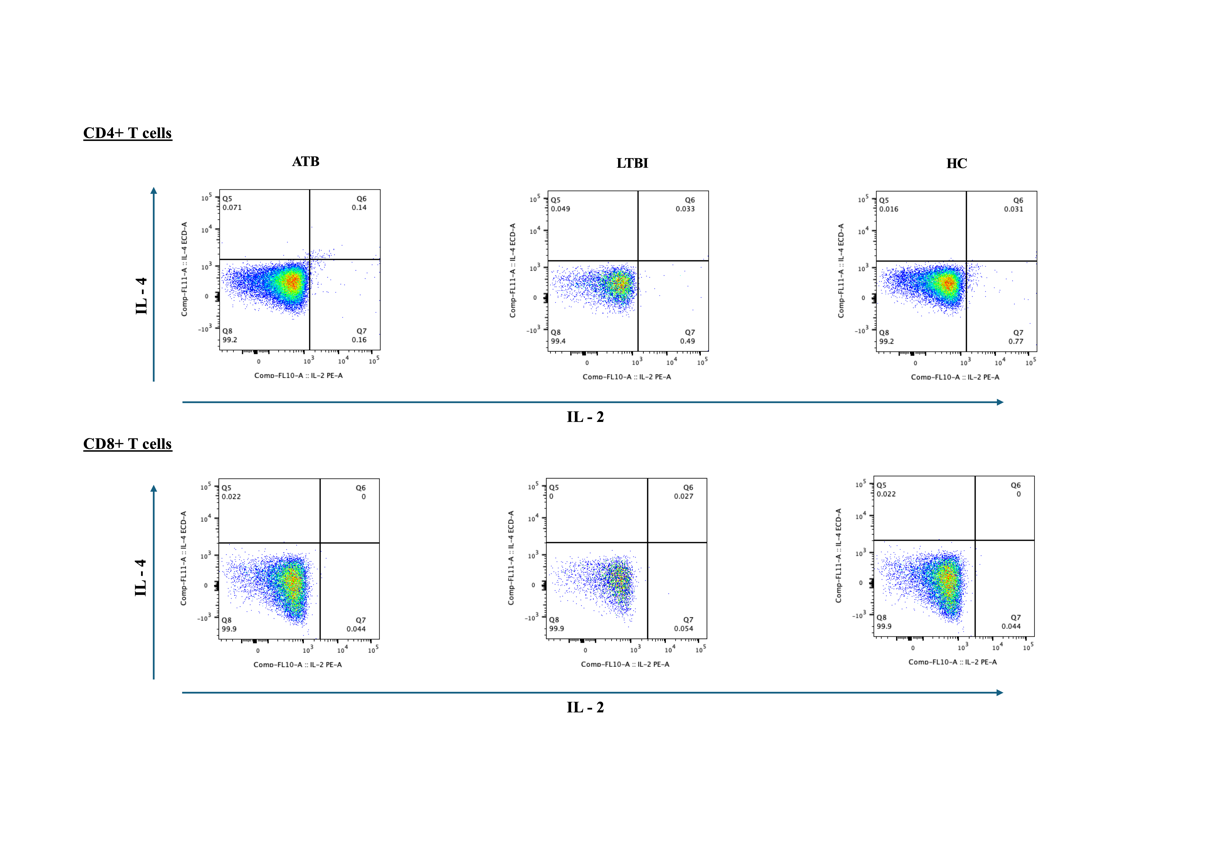


**Figure S.9. Representative flow cytometry plots showing IL-4 and IL-2 production in CD4+ and CD8+ T cells among ATB, LTBI and HC groups.** The top row represents NK cells, white the bottom row represents NKT cells. IL-4 expression is shown on the y-axis and IL-2 expression is shown on the x-axis. Quadrants indicate different cytokine-producing populations. Q1(IL-4 + IL-2 -), Q2 (IL-4 + IL-2 +), Q3 (IL-4 - IL-2 +) and Q4 (double negative). Data illustrate differences in cytokine production across the groups.


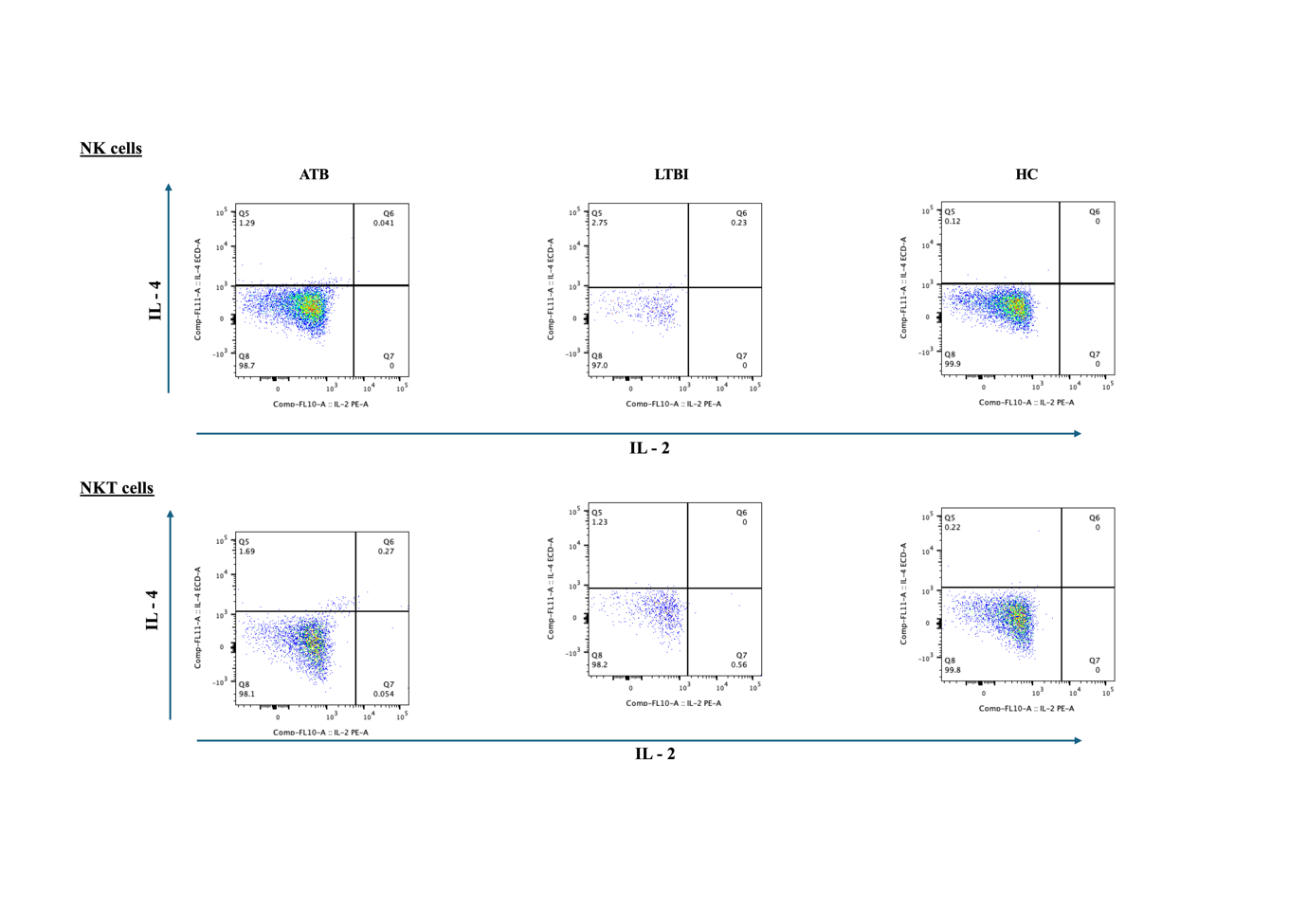


**Figure S.10. Representative flow cytometry plots showing IL-4 and IL-2 production in NK and NKT cells among ATB, LTBI and HC groups.** The top row represents NK cells, white the bottom row represents NKT cells. IL-4 expression is shown on the y-axis and IL-2 expression is shown on the x-axis. Quadrants indicate different cytokine-producing populations. Q1(IL-4 + IL-2 -), Q2 (IL-4 + IL-2 +), Q3 (IL-4 - IL-2 +) and Q4 (double negative). Data illustrate differences in cytokine production across the groups.


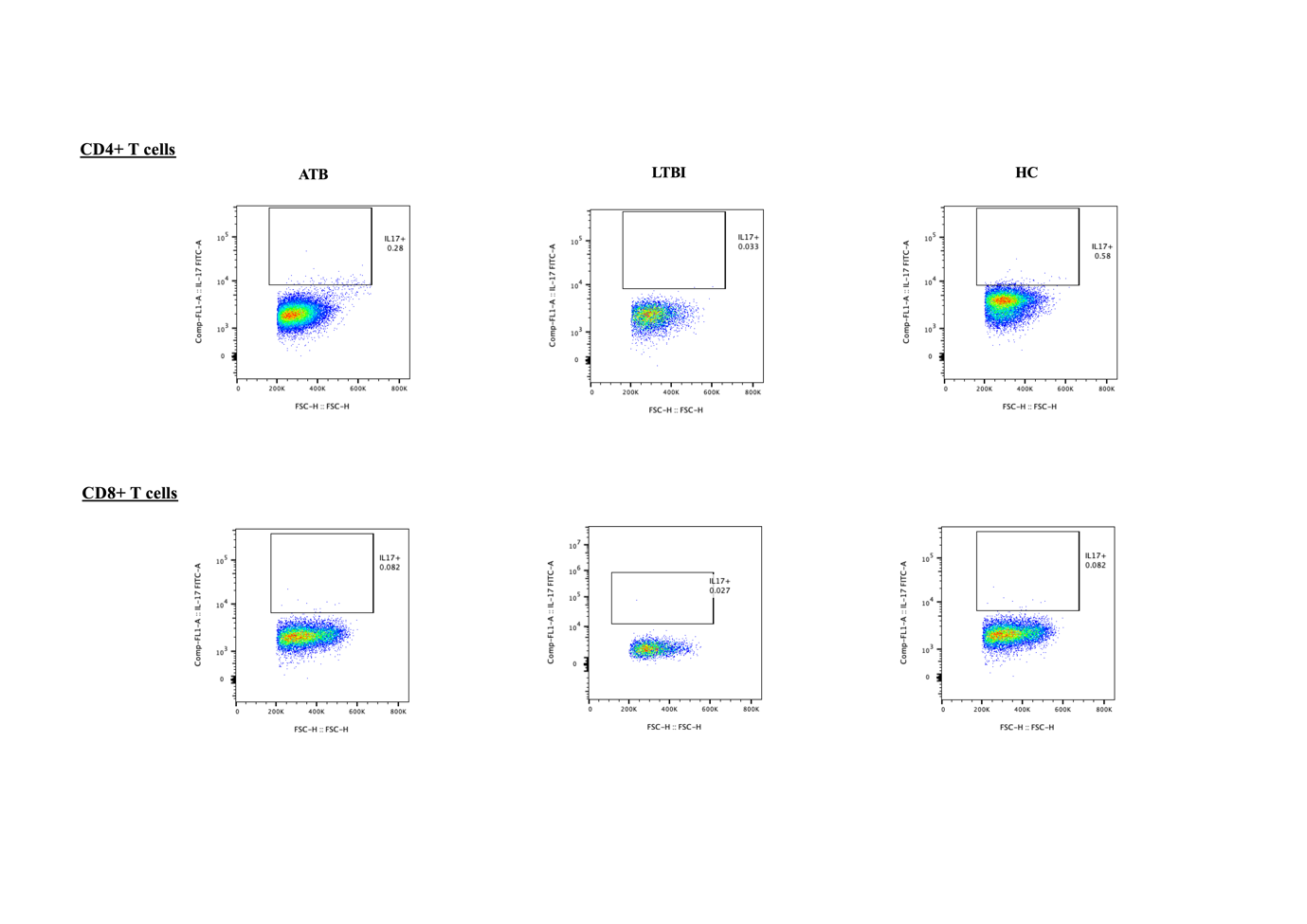


**Figure S.11. Representative flow cytometry plots showing IL-17 in CD4+ and CD8+ T cells among ATB, LTBI and HC groups.** The top row represents CD4+ T cells, white the bottom row represents CD8+ T cells. Data illustrate differences in cytokine production across the groups.


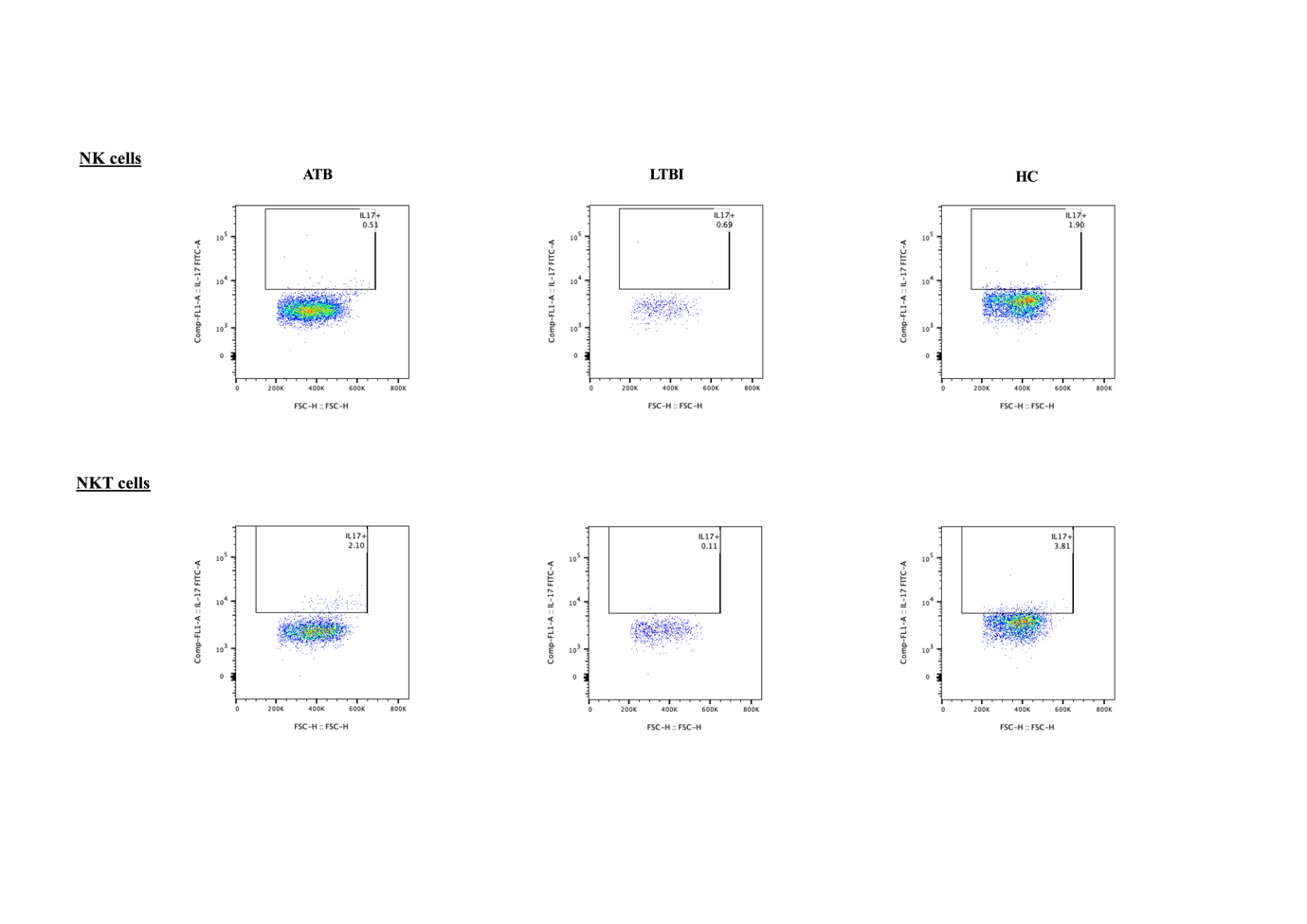


**Figure S.12. Representative flow cytometry plots showing IL-17 in NK and NKT cells among ATB, LTBI and HC groups.** The top row represents NK cells, white the bottom row represents NKT cells. Data illustrate differences in cytokine production across the groups.

**Figure S.13. Group-specific correlation between MGIA outcome (log 10 cfu) and antigen-specific CD4⁺ IFN-γ responses following PPD stimulation in (A) HC, (B) LTBI and (C) ATB. Scatter plots show regression lines with 95% confidence intervals. Each dot represents one participant. Spearman’s correlation coefficient (r) and p-values are shown for each comparison.**

**Figure S.14. Group-specific correlation between MGIA outcome (log 10 cfu) and antigen-specific CD4⁺ IL-4 responses following PPD stimulation in in (A) HC, (B) LTBI and (C) ATB. Scatter plots show regression lines with 95% confidence intervals. Each dot represents one participant. Spearman’s correlation coefficient (r) and p-values are shown for each comparison.**

**Figure S.15. Group-specific correlation of MGIA outcome (log 10 cfu) and antigen-specific CD8⁺ TNF-α responses following PPD stimulation in in (A) HC, (B) LTBI and (C) ATB. Scatter plots show regression lines with 95% confidence intervals. Each dot represents one participant. Spearman’s correlation coefficient (r) and p-values are shown for each comparison.**
